# Supplementary material for: Do powerful CEOs matter for earnings quality? Evidence from Bangladesh
Source: PLoS One. 2023 Jan 20;18(1):e0276935. doi: 10.1371/journal.pone.0276935 (PMC9858436; doi:10.1371/journal.pone.0276935)
Supplement: Attachment — Submitted filename: Manuscript.docx [file pone.0276935.s001.docx]

**Do powerful CEOs matter for earnings quality? Evidence from Bangladesh**

**H M Arif^1^, Mohd Zulkhairi Mustapha^1^*, Azlina Abdul Jalil^1^**

^1^Department of Accounting, Faculty of Business and Economics, University of Malaya, Kuala Lumpur, Malaysia.

*****Corresponding author, E-mail: [zulkhairi@um.edu.my](mailto:zulkhairi@um.edu.my) (MZM)

**Abstract**

This study investigates the effects of powerful Chief Executive Officers (CEOs) on earnings quality in a setting where CEOs are so dominating, they exercise their influence over corporate regulatory bodies. Using a 10-year longitudinal data for the period 2010 to 2019 and 1,395 firm-year observations from listed non-financial firms in Bangladesh, we found that CEOs political power and CEOs with high structural and expert power have a significant detrimental effect on earnings quality. Whereas ownership and prestige power have an insignificant impact on earnings quality. These powerful CEOs use accrual and real activities manipulation techniques together to manage the earnings. This study uses system generalized method of moment estimates for estimation purpose and the results remain robust when alternative earnings quality proxies are used. Taken together our results suggest that CEOs’ political duality (i.e., serving simultaneously as a member of parliament and a CEO) should be restricted and CEOs’ tenure should be limited to a reasonable period. This study enriches the existent literature by furnishing empirical support, including political and prestige power.

**Introduction**

Whether or not powerful CEOs are beneficial or detrimental for earnings quality is an unresolved issue in the academic literature and policy formulation. Proponents of powerful CEOs argue that power aligns CEOs’ interests with other stakeholders by reducing their myopic practices of misreporting earnings to achieve immediate financial targets [1]. Alternatively, other agency theorists posit that excessive power alienates the financial interests of CEOs and general shareholders [2, 3]. It underpins CEOs’ position at the apex of a concern by impairing the board’s supervising effectivity [4]. The practical resonances of this theoretical debate are also evidenced in empirical findings. A detailed review of studies closely associated with the present study reveals that existing studies present inconclusive findings. Some earlier studies report a negative consequence of powerful CEOs on the quality of earnings [5-10] whereas other studies show a positive association [1, 11]. This inconsistency in findings suggests that there is merit in investigating this relationship.

Further, we argue that the existing investigations, that attempt to identify the impact of powerful CEOs on earnings quality [5, 8, 12] consider only a portion of structural (CEO pay slice) or ownership power (CEO equity incentives) and focus on the US (United States) market. These studies ignore other fundamental sources and elements of power. But the power that is also treated as uneven control of valuable resources in social relationships [13, 14] is much more consequential in emerging economies. The US wealth research firm, Wealth-X recently revealed that emerging nations are experiencing the world’s fastest growth in millionaire population [15]. Yet, these nations also suffer from substantial income inequality. Income inequality in developed economies is largely a function of wage disparity. Due to this, several of the prior studies focused on the CEO pay gap with other top executives as a measure for CEO power as this seemed meaningful for the US context. But in emerging economies, income inequality is an all-pervasive inequality of opportunity. Thus, there is a need to extend the existing literature in the setting of emerging economies.

Recently a few studies [16 -18] focused on the aftermath of powerful CEOs on earnings quality. Our research differs from these studies in numerous ways. First, our study focused on CEOs’ political and prestige power in addition to the structural, ownership, and expert power. While Shiah-Hou [18] focused on structural, ownership, and expert power, Sani, Latif, and Al-Dhamari [17] focused on the ownership, expert, and politically connected board and Le, Kweh, Ting, and Nourani [16] mainly focused on ownership and expert power. As a result, there is a knowledge gap regarding the effect of CEO political power and prestige power in extant literature. Our study filled that gap. Second, our study offers empirical documentation from an emerging economy characterized as weak regulatory atmosphere. But Shiah-Hou [18] provided evidence from a developed economy with a strong regulatory atmosphere (the United States context). Although Sani, Latif, and Al-Dhamari [17] and Le, Kweh, Ting, and Nourani [16] focused on emerging economy context, Nigeria, and Vietnam respectively, they offered completely contradictory results. Sani, Latif, and Al-Dhamari [17] evidenced an enhancement in the quality of earnings with the increase in CEO power, whereas Le, Kweh, Ting, and Nourani [16] documented a decline in the reported earnings quality. This contradiction increased the thirst for further investigation in an emerging economy context. Finally, from the methodological point of view, Shiah-Hou [18] and Le, Kweh, Ting, and Nourani [16] used the fixed-effect estimator, which ignores the dynamic attribute of the relation between powerful CEOs and earnings quality. Our study addressed this issue of dynamic endogeneity by executing a well-established dynamic panel GMM (Generalized Method of Moments) estimation. This dynamic estimation method also permits to include time invariant effects to check heterogeneity issues. Although Sani, Latif, and Al-Dhamari [17] used the GMM estimation, the GMM estimation could be severely biased in small samples if the estimated standard errors are not robust or corrected [19]. Our study addressed this issue by applying Windmeijer [19] corrected standard errors.

We consider Bangladesh the most suitable emerging economy for investigating the effects of powerful CEOs on the quality of earnings for three reasons. First, Bangladesh has been identified as the world’s fastest-growing ultra-high wealthy population (those with a net asset of $30 million or more) over the past five years (2012-2017) [20]. It is predicted to have the third quickest growth in terms of rich individuals (persons with a net wealth of $1million or greater but less than $30 million) in the world in the next five years (2018-2023) [15] , and it is a country with high-income inequality that recorded Gini coefficient at 0.482 in 2016 compared to 0.388 in 1992 [21]. This indicates the existence of severe asymmetric control of resources. Further, increasing corruption and the selective enforcement of laws [22] make it easy for powerful individuals to carry on opportunism at the price of general shareholders. For instance, the position of Bangladesh is 146 in the corruption perception index 2020, two steps lower than its 2017 position [23].

Second, manipulated corporate earnings reports have become an issue of extreme concern in Bangladesh. For instance, in an interview with a daily newspaper, the chairperson of the Financial Reporting Council (FRC) mentioned that the government recently formed the FRC after the identification of serious manipulation of many firms’ financial disclosures [24]. Due to the material misstatements, thousands of general investors and financial firms that provide credit facilities to the manipulated firms lose their investments in each year. For instance, when the earnings manipulation by GMG Airlines was reported a few thousand shareholders lost BDT 3000 million, and a state-owned commercial bank lost BDT 2470 million [25]. The country is also losing foreign investment in the stock market due to a lack of trust or confidence in listed firms’ financial statements [24].

Third, we contend that powerful CEOs in Bangladesh perform a major role in the systematic change in earnings quality. Our data show that more than half the CEOs of listed non-financial firms are close relatives to board chairs (e.g., son, daughter, brother, sister, husband, or wife). Eventually, these CEOs have extreme control over corporate internal governance. On top of that, the exercise of political power in the corporate environment by CEOs has gained momentum. Since independence in 1971, 11 parliamentary elections have been held in Bangladesh. In the first parliament (1973-1979), businessmen comprised 18% of the parliament’s members, but in the ninth (2009-2014), tenth (2014-2018), and eleventh parliaments, businessmen represented 57%, 59%, and 61% of the members, respectively [26]. Muttakin, Monem, Khan, and Subramaniam [22] argue that political allies to the party in power are the precondition necessary to protect and advance the economic interest of business leaders in Bangladesh. For example, only one pharmaceutical company has become the exclusive distributor of Covishield (Covid-19 vaccine) in Bangladesh. From January to March 2021 the firm distributed 5 million doses of vaccines (out of 30 million doses) and made a net profit of BDT 383.7 million, which is almost 62.5% higher than the same period of the previous year [27]. Ultimately, the firm will gain a net profit of BDT 2310 million from the distribution of 30 million doses. Interestingly, the CEO and the vice-chairman of the company are both parliament members from the ruling party and the vice-chairman also holds the rank of a cabinet minister for his advisory role to the prime minister [28]. It has been alleged that the firm pressured the government not to adopt alternative sources of vaccines [28]. Hence, this study focuses on CEOs’ political power along with the forms included in [29] taxonomy of power.

This research initiates several contributions. First, our investigation beefs up Finkelstein’s [29] typology of power by adding CEO political power as a source of corporate leaders’ strength and providing empiric attestation regarding the impact of CEOs’ political and prestige power on earnings quality. Second, our study shed light on the debate in agency theory regarding the implication of powerful CEOs on earnings quality in emerging economies by clarifying the mixed findings and providing empirical evidence from Bangladesh. Third, our study addressed the issue of dynamic endogeneity, besides heteroscedasticity and serial correlation, that was ignored in most of the earlier studies. We used the system GMM estimation, that provides consistent results and alleviates endogeneity concerns in existing findings. Scholars suggest that neglecting the dynamic endogeneity may create profound consequences on inference accuracy [30].

Fourth, we use another metric, that of elite educational institutions, which is an element of prestige power that differs from Finkelstein [29] and Lisic, Neal, Zhang, and Zhang [31]. Our measurement addresses the shortcomings of previous attempts to include the metric, as those earlier studies identified only universities from the United States as elite and ignored other top-ranked universities from the rest of the world. They also ignored that university prestige or rank is not fixed and varies over the years. Our study considers the top 300 universities in each year ranked by the Times Higher Education World University Overall Rankings as elite educational institutions.

Finally, the results of this study have some crucial policy suggestions. This study offers mainly two policies. First, a restriction on CEOs’ direct political affiliation may improve the corporate regulatory environment and ultimately the reported earnings quality. This will reduce CEOs’ undue pressure over regulatory bodies. Second, a limit on CEOs’ tenure and involvement of one or two top executives other than the CEO in the corporate board may significantly improve internal corporate governance. Their consequent involvement in strategic decision-making will minimize CEOs’ autocracy.

**Corporate governance regulations in Bangladesh**

The first edition of Corporate Governance Guidelines (CGG) was released by the Securities Commission of Bangladesh in 2006. Yet, the compliance with the guidelines was not mandatory for listed firms [32]. After a massive stock market crash in 2010, the commission amended the CGG in 2012; it contained 95 conditions under seven headings and one annexure [33]. This time compliance with the guidelines was mandatory. In 2018, the commission replaced the CGG with the Corporate Governance Code (CGC) which contains 166 conditions under nine headings and three annexures [34].

Although the CGC has expanded almost 75 percent in terms of the conditions it enforces, it provides enough space for CEOs to exercise extreme power. For instance, both the latest code and the previous CGG restrict CEO duality, but most nonfinancial firms follow this condition in the form but not in the substance. Our data shows that 52% of our sample firms have CEOs and board chairs from the same family and 77% of sample firms have the CEO as the only top management representative who sits on the board. Furthermore, the code is silent regarding the maximum number of committees of a board upon which a CEO can be a member, the maximum tenure of office, and CEOs’ political affiliations. Our data also reveals that each CEO possesses on average 8.05% of their firm’s total equity and their family members possess on average an additional 18.28%; CEOs adopt the position of CEO on average for 11 years; and each CEO secures directorship on 8 other corporate boards. Corporate boards include both public and private limited firms.

**Review of literature**

Power is treated as the capability of a person to enforce his or her desire and influence others’ behavior [29]. Finkelstein [29] classified organizational power into four clusters. These are structural, expert, ownership, and prestige power. In addition to that our study also considers CEOs’ political connection as a key source of power in the emerging economy context. This study views the problem of poor-quality financial reporting via the lens of agency theory (Type II agency problem). In Bangladesh, where a large portion of the non-financial listed firms are family-dominated [35] and more than half the CEOs are family CEOs, firms experience greater Type II agency conflicts (disputes of interests within controlling inside shareholders and minority investors) than Type I. These CEOs act as representatives of other family members in the board, take opportunistic decisions,and extract personal gains through channels such as excessive compensation, special dividends, advantageous related-party transactions, and sometimes even by gaming the system established to monitor the interests of dispersed investors [36]. Eventually, the Type II agency conflicts reduce the earnings quality [37].

**CEO structural power and earnings quality**

A part of CEO power stems from the formal position the CEO holds within a firm. Scholars argue that a manager with a legitimate right to exert influence is influential [14, 29] as it allows CEOs substantial discretion over the allocation of resources [13]. However, whether the excessive structural power of the CEOs is beneficial or harmful for firms is an unsolved question. Empirical studies show both positive and negative impacts on firm financial reporting quality. For example, Hu and Gan [38] noted that CEOs’ greater structural power enhances the firms’ internal control quality. Tee [39] in the Malaysian context mentioned that Powerful CEOs boost the quality of accrual income. Also, Meo, Lara, and Surroca [1] and Seifzadeh, Rajaeei, and Allahbakhsh [40] identified an inverse tie within entrenched managers and income distortion. Contrarily, Shiah-Hou [18] and Baker, Lopez, Reitenga, and Ruch [7] mentioned that CEOs’ greater structural power raises the accrual earnings management. Koo and Kim [5] also identified an increase in firms’ opacity with the increase in CEOs’ structural power. Also, Nuanpradit [9] and Bouaziz, Salhi, and Jarboui [41] documented a supportive nexus within CEO duality and accrual earnings manipulation. Finally, Mande and Son [10] mentioned that high structural power increases the likelihood of beating or meeting market experts’ forecasts. So, there is a lack of abstract knowledge regarding the consequence of high structural power of CEOs on earnings quality in the extant literature. This study expects that excessive structural power will pave the way for CEOs to dominate boards and create pressure on other top executives to harvest short-run stock market gains through manipulating earnings. In Bangladesh, where businesses face a significant lack of regulatory predictability, with powerful business leaders involved in serious corruption [23], there is a favorable environment for abusing excessive power. Therefore, we conjecture the following hypothesis:

Hypothesis 1: CEOs with greater structural power will have a detrimental effect on earnings quality

**Ownership power of CEOs and earnings quality**

Scholars argue that managerial shareholdings reduce board influence and a manager with a significant shareholding will be more dominant than a manager without such ownership [29, 42] . Agency theorists argue that ownership assists to conform the interests of principals and agents [2]. As per this argument, CEO stock ownership should reduce earnings manipulation [43]. However, the excessive share ownership to CEO may also intensify the Type II agency conflicts that ultimately decreases the earnings quality [37, 44, 45]. Extent literature that focused on CEOs’ shareholdings and earnings quality has found inconclusive results. Several studies mentioned that CEO ownership power enhances earnings quality [12, 17, 18, 43, 46]. However, several studies evidence that CEO ownership power reduces internal control quality [38] and intensifies earnings fabrication [8, 47, 48]. Considering the ownership structure in Bangladesh where more than 70% of firms are controlled by family members [35, 49] (a signal of severe Type II agency problems), this study expects to get a negative association within CEO ownership power and earnings quality. So, this study propose the following hypothesis:

Hypothesis 2: CEOs with greater ownership power will have an unfavorable effect on earnings quality

**CEO expert power and earnings quality**

Finkelstein [29] defines expertise as the capability to deal with environmental contingencies. Following Finkelstein [29], this study focuses on CEOs’ functional expertise. This study considers CEOs’ tenure and professional degrees as indicators for functional expertise. Scholars argue that CEOs’ power and the probability of managerial opportunism increase over time [1]. Furthermore, CEOs with accounting expertise know the underlying process of preparing financial statements and the loopholes available in the accounting rules and standards. Consequently, they may find it easy to manipulate a number to share a positive picture of the firm’s financial health. The extant literature provides mixed findings regarding CEOs’ expert power and earnings quality. Several studies mentioned that expert CEOs are fundamental for a better earnings quality [11, 17, 50, 51]. However, other studies suggest that expert CEOs reduce firms’ reported earnings quality [16, 18, 38, 52-54]. Further, Ferramosca and Allegrini [55] documented an insignificant consequence of expert CEOs on earnings quality. Based on the contention that managerial expertise augments managerial power [29, 53] and that power eventually stimulate the desire to manage earnings [53], this study posits that CEOs with greater expertise will have an adverse consequence on earnings quality. Accordingly, our hypothesis is as follows:

Hypothesis 3: CEOs with greater expert power will be negatively associated with earnings quality

**Prestigious CEOs and earnings quality**

Prestige is treated as the respect one possesses in the eyes of others [13]. Scholars suggest that prestige intensify managerial power by absorbing the uncertainty from institutional environment [13, 29]. Further, prestige power induces CEOs to take risky initiatives, since prestigious CEOs’ social ties help to protect their job and improve their chances of re-employment after their turnover for failed risky strategies [56, 57]. Empirical investigations closely associated with our study provide very scant and inconclusive findings about the implication of CEOs with high prestige on earnings quality. A few studies evidence that CEOs with greater connections maintain a high quality of accrual earnings with minimal fraudulent incidents [58, 59]. Still, other investigations report a positive relation within the well-connected CEOs and earnings fabrication [57, 60]. These minimal and inconclusive findings demand more enquiries as to the effect of CEOs prestige power on earnings quality. We expect that as CEOs with high prestige power sit on numerous corporate boards and maintain strong social networks, it is easy for them to manipulate revenues and expenses if required. Therefore, this study posits the following hypothesis:

Hypothesis 4: CEOs with greater prestige power will have a negative outcome on earnings quality

**CEO political power and earnings quality**

Scholars argue that CEOs political affiliation makes it easy to access financial resources and get favorable regulatory treatment [61]. Hence, the political connection is associated with improved firms’ performance. However, some other studies suggest that CEOs’ political likings may weaken the effectivity of the managerial supervision system and curtail the chances of politically linked CEOs being sacked [62]. Also, the political connections work as a secured shelter against punishments for low-quality accounting information [63]. Even in some cases these CEOs are not penalized considering that it may devalue the political party’s image. Eventually, these CEOs become entrenched on the board and other top executives, and they become simply reckless about the quality of information they disclose. Extent literature provides very scant and inconclusive knowledge regarding this issue. Previous investigations mainly considered politically connected boards, not exactly the politically connected CEOs. Some studies show that firms with political association are attached to better earnings informativeness [64], better firm accomplishment [22], and improved value of entities [65]. In contrast, some other studies identified poor quality earnings in politically linked firms [61, 63, 66]. Even, Liu, Li, Zeng, and An [67] documented an inverse U-shaped association within the politically connected firms and transparency in accounting. These indeterminate findings urge further investigation about the politically linked CEOs’ effect on earnings quality. We anticipate that in an emerging economy like Bangladesh, where the competition to access resources is very high and enforcement of laws is selective, CEOs’ political connections stimulate them to manipulate earnings to show better firm achievements. Hence, we postulate the following hypothesis:

Hypothesis 5: CEOs with political power will have an adverse consequence over earnings quality

**Research approach**

**Sampling and data**

This research utilizes a ten-year data set from 165 firms enlisted in the Dhaka stock exchange over the years 2010 to 2019. As of June 30, 2019, a sum of 317 firms are enlisted in the Dhaka Stock Exchange [68]. First, we excluded firms in the bank, insurance, and other financial sectors because these firms are subject to special regulatory treatments and they have specific accounting practices [69, 70]. Next, we excluded the firms in the industry with less than 8 business entities and with insufficient financial data. An ultimate sample size of 1,395 observations from 165 firms is exposed in Table 1.

**Table 1.** **Sample selection**

| Total Firms Listed on Dhaka Stock Exchange on June 30, 2019 | 317 |
| --- | --- |
| Less Banks, Insurance and Financial Institutions | 102 |
| Less firms in the industry with less than 8 firms | 21 |
| Less firms with insufficient financial data | 29 |
| Number of firms treated as final sample | 165 |
| Number of observations | 1395 |

We used the audited financial statements to get financial data of our sample firms, that have been published in their annual reports, and from the database S&P Global Market Intelligence. This study finds non-financial data related to different dimensions of CEO power mainly from publicly available annual reports, prospectuses, and websites of the sample firms. To identify CEOs’ political connections, this study obtained data from the websites of the Bangladesh election commission and Bangladesh parliament in addition to annual reports.

**Earnings quality measures**

To measure earnings quality, compliant with Hope, Thomas, and Vyas [71] we apply two of the most popular proxies of accrual income. Our initial proxy of accrual quality is derived using the revised edition of the Dechow and Dichev [72] model. For each year and industry, equation 1 is appraised using a minimal of 8 observations. Each of the constituents in this equation is scaled by the lag total assets.

| ${TAC}_{it}$ | = | $\gamma_{0}+\gamma_{1}{CFO}_{i,t-1}+\gamma_{2}{CFO}_{it}+\gamma_{3}{CFO}_{i,t+1}+\gamma_{4}\Delta{Rev}_{i,t}+\gamma_{5}{PPE}_{it}+$ |
| --- | --- | --- |
|  |  | $\gamma_{6}{DCFO}_{it}+\gamma_{7}{CFO}_{it}\times{DCFO}_{it}+\epsilon_{i,t}$ (1) |

Where:

| ${TAC}_{it}$ | = | total current accruals calculated as ($\Delta{CA}_{it}-\Delta{CL}_{it}-\Delta{Cash}_{it}+\Delta{Debt}_{it})$ |
| --- | --- | --- |
| $\Delta{CA}_{it}$ | = | yearly variation in total current assets |
| $\Delta{CL}_{it}$ | = | yearly variation in total current liabilities |
| $\Delta{Cash}_{it}$ | = | yearly variation in total cash |
| $\Delta{Debt}_{it}$ | = | yearly variation in total current debt |
| ${CFO}_{it}$ | = | represents cash ﬂows from operating activities |
| $\Delta{Rev}_{i,t}$ | = | yearly variation in sales revenues |
| ${PPE}_{it}$ | = | represents fixed assets (on a gross basis) |
| $DCFO$ | = | 1 for negative cash flows from operation*,* and 0 otherwise |

Discretionary total current accruals (DisTAC) are the residuals from equation 1. Following Hope, Thomas, and Vyas [71], this study first generates the absolute values of these residuals and then multiplies these absolute values by -1. Thus, the bigger value of DisTAC, the better the quality of accrual income.

Our second proxy is derived applying the performance-reconciled model of discretionary accruals recommended by Kothari, Leone, and Wasley [73]. For each year and industry, equation 2 is assessed using a minimal of 8 observations. Each of the constituents in this equation is scaled by the lag total assets.

| ${TA}_{it}$ | = | $\gamma_{0} + \gamma_{1}\left( 1/{{Assets}_{i,t-1}} \right)+ \gamma_{2} \left( \Delta{REV}_{i,t} \right)+\gamma_{3}{PPE}_{i,t} +$ |
| --- | --- | --- |
|  |  | $\gamma_{4}{RoA}_{i,t} + \epsilon_{i,t}$ (2) |

Where:

| $TA$ | = | total accruals measured as$\left( \Delta{CA}_{it}-\Delta{CL}_{it}-\Delta{Cash}_{it}+\Delta{Debt}_{it}-{DEPN}_{i,t} \right)$ |
| --- | --- | --- |
| ${DEPN}_{it}$ | = | represents annual reduction in tangible and intangible assets |
| $PPE$ | = | the worth of fixed assets (on a net basis) of a firm |
| $RoA$ | = | the proportion of net operating income to average assets |

Discretionary total accruals (DisTA) are the residuals from equation 2. As per Hope, Thomas, and Vyas [71], we first generate the absolute values of these residuals and then multiplies these absolute values by -1. Therefore, the larger value of DisTA, the better the quality of accrual income.

**Measures of CEO power dimensions**

**Structural power**

This study considers three elements, CEO’s board committees, CEO duality, and CEO only insider, to measure CEO structural power. First, the variable CEO’s board committees gets the score of 1 if a CEO’s presence in corporate board committees is above or same as the sample median, and 0 else [74]. Next, CEO duality gets a score of 1 if board president and CEO is the same person, and 0 else [31]. Finally, CEO only insider gets the score of 1 if CEO is the only person who represents top management in board meetings, and 0 else [75]. This study then generates CEO structural power (*Strct_pwr*) by adding CEO duality, CEO only insider, and CEO’s board committees.

**Ownership power**

To measure CEO ownership power, this study considers two proxies: CEO’s stock ownership and CEO’s family ownership (excluding the CEO’s portion, to avoid any collinearity) [29]. CEO ownership power (*Own_pwr*) gets the score of 1 if the ownership proportion of a CEO or his or her family members (excluding CEO’s portion) is above or same as the sample median, and 0 else.

**Expert power**

CEO expert power (*Expt_pwr*) gets the score of 1, if a CEO’s years of experience are higher than or same as the sample median or if the CEO possesses a professional degree (CA or CMA or CFA), and 0 else.

**Prestige power**

Following Finkelstein [29] to measure CEOs’ prestige power this study considers three elements of prestige power. These are CEOs’ corporate board membership, nonprofit board membership, and elite education (educational background). Corporate board membership gets the score of 1, if the number of corporate boards a CEO serves on is above or same as the sample median, and 0 otherwise. Nonprofit board membership gets the score of 1 if the number of nonprofit boards on which a CEO serves is above or same as the sample median, and 0 otherwise. Finally, to identify elite educational institutions, this study follows the university rankings by the Times Higher Education. This study treats the top 300 universities each year as elite educational institutions. This is because the government of Bangladesh accepts the top 300 universities by Times Higher Education for offering the most prestigious Prime Minister Fellowship [76]. Elite education gets the score of 6 (most prestigious) if a CEO graduated from a university that ranked within 1-50; 5 if the university ranked within 51-100; 4 if the university ranked within 101-150; 3 if the university ranked within 151-200; 2 if the university ranked within 201-250; 1 (least prestigious) if the university ranked within 251-300; and 0 otherwise. This study then generates CEO prestige power (*Prtge_pwr*) by adding corporate board membership, nonprofit board membership, and elite education.

**Political power**

CEO political power (*Polit_pwr*) gets the score of 1 if the CEO, at some point between 2010 and 2019, was a minister, a member of parliament, a government official, held a top post in the ruling party, or was a close relative to a politician, and 0 else.

**Control variables**

To get a precise effect of our independent variables, several firm specific variables are controlled that previously shown to influence earnings quality. These include firm size (Firm_siz), leverage (Levge), growth in revenues (Growth_Rev) [71]; return on assets (RoA) [77]; Big 4 auditor (Big 4) [78]; negative earnings (NegEarn), firm age (AgE) [35]. Finally, to manage the effect of industry and year, year and industry dummies are added in all estimates [71, 77].

**Estimation method and model**

As the panel data set for this study is dynamic, we opted for the two-step system GMM estimations. Prior studies mentioned that the dynamic panel GMM estimator yields consistent outcomes in the existence of dynamic endogeneity, heterogeneity, and simultaneity [79, 30]. This study executes the xtabond2 command introduced by Roodman [80] for estimating the following econometric model. In all estimations Windmeijer’s [19] corrected standard errors are applied to secure the robustness of findings. All continuous variables have been winsorized at the 1% and 99% level to neutralize the effect of outliers in our data set. Statistical software STATA 14 has been used for analysis purpose.

**Model 1**

| ${EQ}_{it}$ | = | $\gamma{EQ}_{it-1}+\alpha_{1}{Strct\_pwr}_{it}+$ $\alpha_{2}{Own\_pwr}_{it}+\alpha_{3}{Expt\_pwr}_{it}+$ |
| --- | --- | --- |
|  |  | $\alpha_{4}{Prtge\_pwr}_{it}+\alpha_{5}{Polit\_pwr}_{it}+\alpha_{6}{Firm\_siz}_{it}+\alpha_{7}{Levge}_{it}+$ |
|  |  | $\alpha_{8}{RoA}_{it}+\alpha_{9}{AgE}_{it}+\alpha_{10}{NegEarn}_{it}+ \alpha_{11}{Big 4}_{it}+\alpha_{12}{Growth\_Rev}_{it}+$ |
|  |  | $\eta_{t}+\varphi_{i}+\upsilon_{it}$ (3) |

Where:

| ${EQ}_{it}$ | = | denotes earnings quality proxies( TA & TCA) |
| --- | --- | --- |
| ${EQ}_{it-1}$ | = | means prior year value of the respective earnings quality proxy |
| *Strct_pwr* | = | structural power (CEO duality +insider + board committes) |
| *Own_pwr* | = | ownership power (CEO ownership & family ownership) |
| *Expt_pwr* | = | expert power (experience year or professional degree) |
| *Prtge_pwr* | = | prestige power ( CEO multiple directorships both in other companies or non profit + elite education) |
| *Polit_pwr* | = | political power ( either CEO is minister, member of parliament, government officer or close relationship with politician) |
| *Firm_siz* | = | total assets of a firm (in natural log form) |
| *Levge* | = | proportion of total debt to total assets |
| *RoA* | = | proportion of net operating income to average assets |
| *AgE* | = | total years since the company is founded (in natural log form) |
| *NegEarn* | = | 1 if the firm records a net loss in a year, and 0 else |
| *Big 4* | = | 1 if a firm’s financial reports are attested by a Big 4 audit firm, and 0 else |
| *Growth_Rev* | = | percentage increase in revenues from the immediate past year |
| $\eta_{t}$ | = | time-specific effect |
| $\varphi_{i}$ | = | industry-specific effect |
| $\upsilon_{it}$ | = | the error term |

# Results

# Univariate and bivariate analysis

Table 2 displays the descriptive stats concerning different dimensions of the CEO power, selected financial variables, and earnings quality proxies included in our empirical analyses. Table 2 shows that structural power has a minimal score of 0, maximum 3, with a median figure of 2. Ownership, expert, and political power are treated as binary variables. Prestige power varies between 1 to 8 with a median score of 2. Our data shows that out of 1,395 observations, 77 %of the observations have CEOs with high ownership power. While this rate is 55% and 25% for expert and political power respectively-21% of the observations have been audited by a Big 4 auditor which is smaller than that of other emerging economies. For example, Chihua, Tseng, and Chen [53] mentioned that on average 73% of Taiwanese firms have been audited by a Big 4 auditor. Whilst this portion is 62% for listed firms in Pakistan [81]. Two of our main accrual earnings quality proxies (DisTAC and DisTA) show that firms on average adjust accruals of 9.8% and 15.7% of total assets for DisTAC and DisTA respectively. Thus, the reported earnings are higher than actual due to the earnings management practices. Compared to other developed and developing economies the earnings quality of Bangladeshi listed firms is 2 times below than that of the U.S. companies [71] and 4% lower than that of Taiwanese listed firms [16].

**Table 2.** **Descriptive statistics**

| **Variables** | **Obs.** | **Mean** | **SDev.** | **Mini.** | **50%** | **75%** | **Maxi.** |
| --- | --- | --- | --- | --- | --- | --- | --- |
| Strct_pwr | 1395 | 1.73 | .532 | 0 | 2 | 2 | 3 |
| Own_pwr | 1395 | .771 | .420 | 0 | 1 | 1 | 1 |
| Expt_pwr | 1395 | .546 | .498 | 0 | 1 | 1 | 1 |
| Prtge_Pwr | 1395 | 2.248 | .489 | 1 | 2 | 2 | 8 |
| Polit_pwr | 1395 | .253 | .435 | 0 | 0 | 1 | 1 |
| NegEarn | 1395 | .091 | .288 | 0 | 0 | 0 | 1 |
| Big 4 | 1395 | .209 | .407 | 0 | 0 | 0 | 1 |
| Levge | 1395 | .512 | .474 | .003 | .459 | .658 | 7.516 |
| AgE (log of firm’s age) | 1395 | 3.024 | .610 | 1.386 | 3.045 | 3.497 | 4.234 |
| Growth_Rev | 1395 | 11.695 | 25.380 | -55.031 | 8.291 | 22.017 | 73.605 |
| Firm_siz (log of total assets) | 1395 | 21.673 | 1.519 | 17.197 | 21.651 | 22.583 | 26.022 |
| RoA | 1317 | .055 | .091 | -1.073 | .045 | .085 | .626 |
| DisTA | 1317 | -.157 | .253 | -4.412 | -.098 | -.045 | 0 |
| DisREV | 1317 | -.040 | .081 | -1.995 | -.023 | -.012 | 0 |
| DisTAC | 1252 | -.098 | .193 | -4.687 | -.061 | -.025 | 0 |
| Ab_P_C | 1217 | .803 | .720 | -.031 | 0.603 | 0.952 | 4.200 |

## Table 3 shows the independent variables’ correlation matrix. The correlation among the individual dimensions of CEO power is very weak, which indicates their distinctiveness and removes the concern of multicollinearity issues for this study.

**Table 3. Correlation matrix**

| Variables | Strct_pwr | Own_pwr | Expt_pwr | Prtge_Pwr | Polit_pwr |
| --- | --- | --- | --- | --- | --- |
| Strct_pwr | 1.000 |  |  |  |  |
| Own_pwr | 0.095* | 1.000 |  |  |  |
| Expt_pwr | 0.080* | 0.265* | 1.000 |  |  |
| Prtge_Pwr | -0.007 | 0.156* | 0.063* | 1.000 |  |
| Polit_pwr | 0.113* | -0.068* | -0.178* | 0.016 | 1.000 |

## * indicate significant at 10% level

## Multivariate analysis

## Earnings quality and dimensions of CEO power

To verify Hypotheses 1, 2, 3, 4, and 5, Model 1 is estimated. Table 4 presents the outcomes of regressing the hypothesized variables onto earnings quality proxies. These results highlight some important insights. First, the statistically significant coefficients of the variables Lag DisTAC and Lag DisTA, indicate that there is a correlation between past and present values of earnings quality proxies. This provides the support of our choice of a dynamic estimation method, two-step system GMM, for this study.

Second, Hypothesis 1 states that CEOs with greater structural power are adversely sticked to the accrual earnings quality. The estimation outcomes of Model 1 in Table 4 demonstrate that *Strct_pwr* ($\alpha_{1} =$-0.041, *p* < 0.01, and $\alpha_{1} =$-0.070, *p* < 0.01) has an inverse and statistically significant effect on both the earnings quality proxies, which supports Hypothesis 1. These findings imply that the existence of CEOs with high structural power is annexed to the reduction in earnings quality by 4.1% and 7% of total assets for the accrual quality proxies DisTAC and DisTA, respectively.

Hypothesis 2 predicts that CEOs with inordinate ownership power are harmful to earnings quality. The results displayed in Table 4 attest that ownership power has an insignificant bearing on either earnings quality proxy, which refutes Hypothesis 2.

Hypothesis 3 states that CEOs with greater expertise will be tied up with lower earnings quality. The statistically significant negative coefficients of *Expt_Pwr* ($\alpha_{2} =$-0.031, *p* < 0.05, and $\alpha_{2} =$-0.061, *p* < 0.05) across both earnings-quality proxies support Hypothesis 3.These results suggest that the presence of more expert CEOs are associated with 3.1% increases in discretionary current accruals (DisTAC), and 6.1% increase of abnormal accruals (DisTA). In other words, CEOs who hold the position for longer tenure or have a professional accounting degree are more involved in earnings manipulation activities in Bangladesh.

Hypothesis 4 predicts that CEOs with greater prestige power will be adversely attached to the quality of accrual income. Results in Table 4 illustrate that the effect of prestige power is insignificant for both proxies, which rejects Hypothesis 4.

Hypothesis 5 states that politically connected CEOs maintain lower quality earnings. The significant and negative coefficients of *Polit_pwr* ($\alpha_{3} =$-0.058, *p* < 0.05, and $\alpha_{3} =$-0.187, *p* < 0.05) across both earnings-quality proxies support Hypothesis 5. These findings offer evidence that the politically connected CEOs are responsible for a 5.8% increment in discretionary current accruals (DisTAC), and an 18.7% surge in abnormal total accruals (DisTA) in listed non-financial firms in Bangladesh.

Results related to control variables show that *RoA* and *NegEarn* are negatively associated with the accrual quality proxies, suggesting that to show better firm performance and to hide actual performance, firms in Bangladesh compromise with earnings quality. Also, the variable *Levge* is oppositely linked with earnings quality, suggesting that with the rise in firms’ debt, earnings quality also reduces. This is logical as this study does not find that *Big 4* (Big 4) audit firms have any significant effect on earnings quality in the Bangladeshi context. However, Firm_siz shows a significantly positive association with earnings quality, indicating that larger firms maintain better quality earnings. Overall, these findings endorse the agency theory stance as powerful CEOs are detrimental to a firm’s earnings quality, especially if they possess excessive structural, expert, and political power.

Finally, the post estimation results of the system-GMM estimates disclose that our estimated models are properly determined as the F-stats are statistically significant at 1% level for both earnings quality proxies. The three main diagnostic results are as follows. First, the probability values of *A.R. (1)* are statistically significant indicating that there is a high first-order autocorrelation in each estimation; however, the probability values for *A.R. (2)* are insignificant, showing the proof of no second-order autocorrelation. Next, the Hansen J-statistics report insignificant p-values, which certify the validity of instruments used in this study. Finally, this study used lower number of instruments as against the number of firms, which keeps these estimates free of the instrument proliferation problem [82].

**Table 4. Dimensions of CEO power and earnings quality:** **System GMM estimates**

| **Variables** | **Pred. Sign** | **DisTAC** | **P-value** | **DisTA** | **P-value** |
| --- | --- | --- | --- | --- | --- |
| Lag DisTAC |  | 0.215*** | 0.002 |  |  |
| Lag DisTA |  |  |  | 0.336*** | 0.001 |
| Strct_pwr | - | -0.041*** | 0.008 | -0.070*** | 0.006 |
| Own_pwr | - | -0.015 | 0.557 | 0.006 | 0.923 |
| Expt_pwr | - | -0.031** | 0.043 | -0.061** | 0.047 |
| Prtge_Pwr | - | -0.006 | 0.249 | 0.001 | 0.852 |
| Polit_pwr | - | -0.058** | 0.039 | -0.187** | 0.033 |
| Firm_siz |  | 0.005** | 0.025 | 0.015** | 0.035 |
| Levge |  | -0.042 | 0.106 | -0.152 | 0.146 |
| RoA |  | -0.518*** | 0.002 | -0.953 | 0.104 |
| AgE |  | 0.005 | 0.537 | -0.035 | 0.168 |
| NegEarn |  | -0.077*** | 0.005 | -0.090 | 0.250 |
| Big4 |  | -0.020 | 0.388 | 0.054 | 0.401 |
| Growth_Rev |  | -0.001 | 0.906 | 0.001 | 0.697 |
| Year and Ind. Dum. |  | Included |  | Included |  |
| No. of firm-years |  | 1034 |  | 1034 |  |
| F-stat |  | 22.95*** | 0.001 | 13.48*** | 0.001 |
| Number of firms considered |  | 165 |  | 165 |  |
| Number of instruments used |  | 122 |  | 122 |  |
| A.R. (1) ρ-val. |  |  | 0.001 |  | 0.014 |
| A.R. (2) ρ-val. |  |  | 0.968 |  | 0.259 |
| Hansen *J* stats p-val. |  |  | 0.884 |  | 0.299 |

GMM estimation with Windmeijer [19] corrected standard errors. Symbols ***, ** and * indicate significant at 1%, 5% and 10%, respectively.

**Tests of robustness**

**Alternate earnings quality proxy**

For assessing the robustness of our main results, we apply an alternate earnings quality proxy, discretionary revenue, as the dependent variable, and estimates Model 1The proxy is extracted from revenue-accrual quality, as proposed by Stubben [83] and adopted from Hope, Thomas, and Vyas [71]. In this measure, receivables accrual is modeled as a function of change in revenues. Specifically, for each industry and year equation 4 is estimated for a minimal of 8 observations. Each of the constituents in this equation is scaled by the lag total assets.

$$\Delta{AcR}_{i,t} = \delta_{0} +\delta_{1}\Delta{Rev}_{i,t}+\in_{i,t} (4)$$

In this equation, $\Delta{AcR}_{i,t}$ stands for yearly variation in accounts receivable, $\Delta{Rev}_{i,t}$ depicts yearly change in sales revenues. Discretionary revenues (DisREV) are the residuals’ absolute values from equation 4. Following Hope, Thomas, and Vyas [71], this study multiplies these absolute values by -1, so as the bigger values of DisREV, the better the quality of earnings.

The findings in Table 5 reveal that structural, expert, and political power have significant and negative coefficients for discretionary revenue (DisREV) at a 5% level. However, CEOs’ ownership and prestige power are insignificantly associated with earnings quality. These results also support Hypotheses 1, 3, and 5, and suggest that CEOs with substantial structural, expert, and political power are associated with sales manipulation that lower the quality of accounting earnings.

**Table 5. CEO power aspects and earnings quality (DisREV)**

| **Variables** | **Pred. Sign** | **DisREV** | **P- value** |
| --- | --- | --- | --- |
| Lag DisREV |  | 0.260*** | 0.001 |
| Strct_pwr | - | -0.019** | 0.033 |
| Own_pwr | - | 0.006 | 0.549 |
| Expt_pwr | - | -0.021** | 0.047 |
| Prtge_Pwr | - | -0.002 | 0.618 |
| Polit_pwr | - | -0.053** | 0.043 |
| Firm_siz |  | 0.006* | 0.068 |
| Levge |  | -0.039 | 0.267 |
| RoA |  | -0.382** | 0.044 |
| AgE |  | -0.019 | 0.212 |
| NegEarn |  | -0.017 | 0.162 |
| Big4 |  | 0.030 | 0.184 |
| Growth_Rev |  | 0.001 | 0.389 |
| Year and Ind. Dum. |  | Included |  |
| No. of firm-years |  | 1034 |  |
| F-stat |  | 6.08*** | 0.001 |
| Number of firms considered |  | 165 |  |
| Number of instruments used |  | 122 |  |
| A.R. (1) ρ-val. |  |  | 0.011 |
| A.R. (2) ρ-val. |  |  | 0.717 |
| Hansen *J* stats ρ-val. |  |  | 0.529 |

GMM estimation with Windmeijer [19] corrected standard errors. Symbols ***, ** and * indicate significant at 1%, 5% and 10%, respectively.

**Additional analysis**

To check whether the powerful CEOs use real transactions management techniques besides accrual earnings management to manipulate earnings, this study also used a real activities management proxy, abnormal production costs (Ab_P_C). This proxy is measured using Roychowdhury [84] model. Particularly, equation 5 is estimated to get the proxy values:

| $\frac{{Prod}_{it}}{{Asst}_{it-1}}$ | = | $\gamma_{0}+\gamma_{1}\left( \frac{1}{{Asst}_{it-1}} \right)+\gamma_{2}\left( \frac{{Sls}_{it}}{{Asst}_{it-1}} \right)+\gamma_{3}\left( \frac{{\Delta Sls}_{it}}{{Asst}_{it-1}} \right)+$ |
| --- | --- | --- |
|  |  | $\gamma_{4}\left( \frac{{\Delta Sls}_{it-1}}{{Asst}_{it-1}} \right)+\varepsilon_{it} (5)$ |

Where ${Prod}_{it}$ is the the summation of cost of products or services sold out and the yearly variation in the value inventory. ${Asst}_{it-1}$ represents worth of assets at the outset of a year. ${Sls}_{it}$ is for sales during a year, $\Delta{Sls}_{it}$ represents yearly change in sales amount, and $\Delta{Sls}_{it-1}$ represents one year lagged change in sales value. Abnormal production costs (Ab_P_C) are the residuals from equation (5). This study then estimates Model 1 to ascertain the consequence of excessive CEO power upon abnormal production costs. This study expects a positive association among the CEOs’ power dimensions and abnormal production costs.

The results in Table 6 show that structural, expert, and political power are significantly (at 5% level) and positively associated with abnormal production costs. But ownership and the prestige show an insignificant effect on Ab_P_C. These results support hypotheses 1, 3, and 5, and suggest that CEOs with high structural, expert, and political power use the technique of producing more products than demands to show better firm performance by scaling down the cost per unit of products sold.

**Table 6. CEO power facets and earnings quality (Ab_P_C)**

| **Variables** | **Pred. Sign** | **Ab_P_C** | **P-value** |
| --- | --- | --- | --- |
| Lag Ab_P_C |  | 0.294* | 0.073 |
| Strct_pwr | + | 0.195** | 0.045 |
| Own_pwr | + | -0.130 | 0.221 |
| Expt_pwr | + | 0.204** | 0.019 |
| Prtge_Pwr | + | 0.014 | 0.667 |
| Polit_pwr | + | 0.315** | 0.030 |
| Firm_siz |  | -0.047** | 0.012 |
| Levge |  | 0.352*** | 0.004 |
| RoA |  | 0.682*** | 0.001 |
| AgE |  | 0.105 | 0.112 |
| NegEarn |  | 0.255** | 0.046 |
| Big 4 |  | -0.050 | 0.622 |
| Growth_Rev |  | -0.001 | 0.427 |
| Year and Ind. Dum. |  | Included |  |
| No. of firm-years |  | 869 |  |
| F-stat |  | 67.65*** | 0.001 |
| Number of firms considered |  | 161 |  |
| Number of instruments used |  | 109 |  |
| A.R. (1) ρ-val. |  |  | 0.036 |
| A.R. (2) ρ-val. |  |  | 0.224 |
| Hansen *J* stats ρ-val. |  |  | 0.767 |

GMM estimation with Windmeijer [19] corrected standard errors. Symbols ***, ** and * indicate significant at 1%, 5% and 10%, respectively.

# Discussion and conclusion

This study has provided evidence from an emerging economy context that the quality of earnings will be reduced if the power is centered only in the hands of a CEO. Employing 1,395 observations from firms listed in Dhaka stock exchange, Bangladesh, our results exhibit that CEOs with greater structural power, considerable expertise, and the politically connected CEOs are involved in inferior financial reporting practices. These CEOs, besides accrual earnings management, apply real activities management techniques to manipulate the reported earnings. However, CEOs with high ownership and prestige power have an insignificant effect on earnings quality. These results remain unchanged when alternative earnings quality proxies are used. These results suggest that powerful CEOs especially with excessive structural and expert power, and politically connected CEOs are detrimental to the interests of residual claimants (specifically for minority stockholders) in emerging economies like Bangladesh.

The outcomes of our study are consistent with agency theory, what states that people are self-interested and will have dispute of interests whenever they aim to involve in any mutual initiatives [85]. Since, in Bangladesh most of the listed firms have a family concentrated ownership structure, this study considers these conflicts of interests mainly between controlling insiders (CEOs and their family members) and non-controlling outside investors (Type II agency conflicts). Considering Bangladesh context Ahmed and Uddin [86] mentioned that general shareholders always identify themselves in a condition of non-controlling investors, with the prime objective to make money quickly by short-term trading. Powerful CEOs and their family members exploit this tendency of general investors by inflating earnings report. So, the Type II agency conflict which suggests lower earnings quality is evidenced in this study.

The findings of our study are logical for several reasons. First, in Bangladesh, political power is considered the most influential power among all power dimensions. Political identity gets the highest priority in getting a license for any shorts of commercial activities and the politically connected business leaders perform almost monopoly business in every industry. Interestingly, although they make a huge amount of profit, they do not care about depriving non-controlling shareholders by manipulating financial statements. They tunnel the resources of a cash cow firm in their different private business projects. Rules and regulations are mainly for managers with low power.

Second, the status of a CEO is itself a root of structural power, and the addition of other elements of dominance (such as being the chair of the board, being the only top management representative in the board, or being a member of different board committees) add stronger layers to the structural power of a CEO [87]. This high level of structural power opens the door for the CEO to create undue pressure over other top executives. Whereas other top executives enjoy very insignificant formal power within the organization compared to CEOs. Powerful CEOs control the pay and promotions of other executives. The recruitments, promotions, bonuses, and firing from the job are not performed formally. These tasks are performed informally, arbitrarily, and with direct interventions by CEOs [88]. Eventually, most of the top executives have higher career concerns relative to the CEOs.

Third, in the corporate governance code of Bangladesh, there is no limit on the tenure of a CEO. As a result, these long-tenured CEOs establish control over internal monitoring mechanisms. In many cases, CEO himself becomes the part of the monitoring mechanism by being a member of the audit committee. So, in most cases, monitoring organ of a firm plays a ceremonial role. This paves the way for CEOs to take self-centered initiatives harmful to minority shareholders.

Fourth and finally**,** regulatory bodies play a silent role regarding the wrongdoing of the powerful CEOs especially politically powerful CEOs. This is because if they take any action against these CEOs, they may even lose their position or maybe shifted to other less important sections of the government [86]. Interestingly, the top positions of corporate regulatory bodies are also filled by explicitly politically affiliated persons with the party in power. As a result, these top officials of regulatory bodies offer preferential treatments to politically connected CEOs, as they follow the same political ideology. Prior studies mentioned that rather than ensuring good governance, in some cases regulatory bodies work as indirect catalysts for bad corporate practices [89]. Further, the lack of skilled, competent, and independent manpower in regulatory bodies is also a prime cause for the poor monitoring system.

# References

1.	Meo FD, Lara JMG, Surroca JA. Managerial entrenchment and earnings management. Journal of Accounting and Public Policy. 2017; 36(5): 399-414.

2.	Jensen MC, Meckling WH. Theory of the firm: Managerial behavior, agency costs and ownership structure. J Finan Econ. 1976; 3(4): 305-60.

3.	Sheikh S. CEO power, product market competition and firm value. Research in International Business and Finance. 2018; 46: 373-86.

4.	Finkelstein S, D'aveni RA. CEO Duality as a Double-Edged Sword: How Boards of Directors Balance Entrenchment Avoidance and Unity of Command. Academy of Management Journal. 1994; 37(5): 1079-108.

5.	Koo K, Kim J. CEO power and firm opacity. Applied Economics Letter. 2019; 26(10): 791-4.

6.	Khanna V, Kim EH, Lu Y. CEO connectedness and corporate fraud. The Journal of Finance. 2015; 70(3): 1203-52.

7.	Baker TA, Lopez TJ, Reitenga AL, Ruch GW. The influence of CEO and CFO power on accruals and real earnings management. Rev Quant Finance Acc. 2019; 52(1):1-21.

8.	Leon L, ChiiShyan K. CEO equity compensation and earnings management: The role of growth opportunities. Finance Research Letters. 2017; 20: 289-95.

9.	Nuanpradit S. Real earnings management in Thailand: CEO duality and serviced early years. Asia-Pacific Journal of Business Administration. 2019; 11(1): 88-108.

10.	Mande V, Son M. CEO Centrality and Meeting or Beating Analysts’ Earnings Forecasts. Journal of Business Finance & Accounting. 2012;39(1‐2):82-112.

11.	Gounopoulos D, Pham H. Financial expert CEOs and earnings management around initial public offerings. The International Journal of Accounting. 2018;53(2):102-17.

12.	Petrou AP, Procopiou A. CEO Shareholdings and Earnings Manipulation: A Behavioral Explanation. European Management Review. 2016;13(2):137-148.

13.	Magee JC, Galinsky AD. social hierarchy: The self‐reinforcing nature of power and status. Academy of Management annals. 2008;2(1):351-398.

14.	Ocasio W, Pozner J-E, Milner D. Varieties of Political Capital and Power in Organizations: A Review and Integrative Framework. Academy of Management Annals. 2020;14(1):303-338.

15.	White V, Shaban M. High Net Worth Handbook 2019. Wealth-X website; 2019. Avaliable from: https://www.wealthx.com/wp-content/uploads/2019/01/Wealth-X_High-Net-Worth-Handbook-2019.pdf

16.	Le HTM, Kweh QL, Ting IWK, Nourani M. CEO power and earnings management: Dual roles of foreign shareholders in Vietnamese listed companies. International Journal of Finance & Economics. 2020. doi: https://doi.org/10.1002/ijfe.2211

17.	Sani AuA, Latif RA, Al-Dhamari RA. CEO discretion, political connection and real earnings management in Nigeria. Management Research Review. 2020;43(8):909-29.

18.	Shiah-Hou S-R. Powerful CEOs and earnings quality. Managerial Finance. 2021. doi: https://doi.org/10.1108/MF-07-2020-0363

19.	Windmeijer F. A finite sample correction for the variance of linear efficient two-step GMM estimators. J Econometrics. 2005;126(1):25-51.

20.	White V, Imberg M, Chesterfield W, Shaban M. World Ultra Wealth Report 2018. Wealth-X website: Wealth-X; 2018 September 5. Available from: https://thehometrust.com/wp-content/uploads/2018/09/Wealth-X_World-Ultra-Wealth-Report_Sept-2018_PDF.pdf

21.	Byron RK, & Parvez, S. Inequality at all-time high. The Daily Star. 2019 May 2019. Available from: https://www.thedailystar.net/frontpage/news/inequality-all-time-high-1745569

22.	Muttakin MB, Monem RM, Khan A, Subramaniam N. Family firms, firm performance and political connections: Evidence from Bangladesh. Journal of Contemporary Accounting & Economics. 2015;11(3): 215-30.

23.	Transparency International. Corruption Perceptions Index 2020. Transparency International website; 2021 January 2021. Available from: https://images.transparencycdn.org/images/CPI2020_Report_EN_0802-WEB-1_2021-02-08-103053.pdf

24.	Habib A. Financial Reporting Council not fully ready. The Daily Star. 2019 March 12; Sect. Business. Available from: https://www.thedailystar.net/business/news/financial-reporting-council-not-fully-ready-1713802

25.	Alo JN. Curious case of GMG loan. The Daily Star. 2018 March 03; Sect. Front Page. Available from: https://www.thedailystar.net/frontpage/curious-case-gmg-loan-1542823

26.	Aktar M, Roy NR, Akram SM, Sultana N, Zurat F. Parliament Watch: 11th Parliament (1st to 5th Session). Transparency International Bangladesh website; 2020 September 30. Available from: https://www.tibangladesh.org/beta3/images/2020/report/PW/PW_Full_Report.pdf

27.	Mohajan S, Kabir A. Beximco makes 77 taka profit on each dose of Covid vaccine. Prothom Alo. 2021 May 02. Available from: https://en.prothomalo.com/business/local/beximco-makes-77-taka-profit-on-each-dose-of-covid-vaccine

28.	Julkarnayeen M, Alam MN, Akter M, Akter T. Tackling Coronavirus Pandemic:

Governance Challenges in COVID-19 Vaccine Management. Transparency International Bangladesh website: Transparency International Bangladesh.; 2021. Available from: https://www.tibangladesh.org/beta3/images/2021/report/COVID_Response_tracking_III_ES_English_080621_.pdf

29.	Finkelstein S. Power in top management teams: Dimensions, measurement, and validation. Academy of Management journal. 1992;35(3):505-38.

30.	Wintoki MB, Linck JS, Netter JM. Endogeneity and the dynamics of internal corporate governance. J Finan Econ. 2012; 105(3):581-606.

31.	Lisic LL, Neal TL, Zhang IX, Zhang Y. CEO Power, Internal Control Quality, and Audit Committee Effectiveness in Substance Versus in Form. Contemporary Accounting Research. 2016;33(3):1199-237.

32.	Khan A, Muttakin MB, Siddiqui J. Corporate Governance and Corporate Social Responsibility Disclosures: Evidence from an Emerging Economy. Journal of Business Ethics. 2013;114(2):207-23.

33.	Bangladesh Securities and Exchange Commission. Revised Corporate Governance Guidelines. Bangladesh Securities and Exchange Commission.; 2012, September. Available from: https://www.sec.gov.bd/slaws/Notification_on_CG-07.8.12-Amended.pdf

34.	Bangladesh Securities and Exchange Commission. Corporate Governance Code. Bangladesh Securities and Exchange Commission.; 2018, June. Available from: https://www.sec.gov.bd/slaws/Corporate_Governance_Code_10.06.2018.pdf

35.	Razzaque RMR, Ali MJ, Mather PR. Real earnings management in family firms: Evidence from an emerging economy. Pacific-Basin Finance Journal. 2016; 40:237-50.

36.	Habib A. Central Pharma directors game the system. The Daily Star. 2018 May 20;Sect. Business. Available from: https://www.thedailystar.net/business/central-pharma-directors-game-the-system-1578886

37.	Srinidhi BN, He S, Firth M. The effect of governance on specialist auditor choice and audit fees in US family firms. The Accounting Review. 2014;89(6):2297-329.

38.	Hu M, Gan S. Life cycle stage effects, CEO power and internal control quality: evidence from listed family firms in China. China Journal of Accounting Studies. 2017;5(2):211-33.

39.	Tee CM. CEO power and audit fees: Evidence from Malaysia. International Journal of Auditing. 2019;23(3):365-86.

40.	Seifzadeh M, Rajaeei R, Allahbakhsh A. The relationship between management entrenchment and financial statement fraud. Journal of Facilities Management. 2021. doi: https://doi.org/10.1108/JFM-02-2021-0026

41.	Bouaziz D, Salhi B, Jarboui A. CEO characteristics and earnings management: empirical evidence from France. Journal of Financial Reporting and Accounting. 2020. doi: https://doi.org/10.1108/JFRA-01-2019-0008

42.	Fang H, Lee J-S, Chung C-P, Lee Y-H, Wang W-H. Effect of CEO power and board strength on bank performance in China. J Asian Econ. 2020; 69:101215. doi: https://doi.org/10.1016/j.asieco.2020.101215

43.	Zhang X, Bartol KM, Smith KG, Pfarrer MD, Khanin DM. CEOs on the edge: Earnings manipulation and stock-based incentive misalignment. Academy of Management Journal. 2008;51(2):241-58.

44.	Anderson RC, Reeb DM. Board composition: Balancing family influence in S&P 500 firms. Administrative science quarterly. 2004;49(2):209-37.

45.	Lansberg IS. Managing human resources in family firms: The problem of institutional overlap. Organizational dynamics. 1983;12(1):39-46.

46.	Duellman S, Ahmed AS, Abdel-Meguid AM. An empirical analysis of the effects of monitoring intensity on the relation between equity incentives and earnings management. Journal of Accounting and Public Policy. 2013;32(6):495-517.

47.	Feng M, Ge W, Luo S, Shevlin T. Why do CFOs become involved in material accounting manipulations? Journal of Accounting and Economics. 2011;51(1-2):21-36.

48.	Leon L, ChiiShyan K. CEO equity compensation and earnings management: The role of growth opportunities. Finance Research Letters. 2017;20:289-95.

49.	Rashid A. CEO duality and agency cost: evidence from Bangladesh. Journal of Management & Governance. 2013;17(4):989-1008.

50.	Oradi J, Asiaei K, Rezaee Z. CEO financial background and internal control weaknesses. Corporate Governance: An International Review. 2020;28(2):119-40.

51.	Saito Y. Are long-tenured CEOs rent seekers? Analysis of cash compensation and post disposal performance. Advances in accounting. 2019;44:95-107.

52.	Altunbaş Y, Thornton J, Uymaz Y. CEO tenure and corporate misconduct: Evidence from US banks. Finance Research Letters. 2018;26:1-8.

53.	Chihua L, Tseng Y, Chen T-K. Top management team expertise and corporate real earnings management activities. Advances in Accounting. 2016;34:117-32. doi: https://doi.org/10.1016/j.adiac.2016.07.007

54.	Priscilla M, Siregar SV. The Effect of Top Management Team Expertise on Corporate’s Accrual and Real Earnings Management. Advanced Issues in the Economics of Emerging Markets: Emerald Publishing Limited; 2020. doi: https://doi.org/10.1108/S1571-038620200000027007

55.	Ferramosca S, Allegrini M. The complex role of family involvement in earnings management. Journal of Family Business Strategy. 2018;9(2):128-41.

56.	Dbouk W, Fang Y, Liu L, Wang H. Do social networks encourage risk-taking? Evidence from bank CEOs. Journal of Financial Stability. 2020;46:100708. doi: https://doi.org/10.1016/j.jfs.2019.100708

57.	Griffin PA, Hong HA, Liu Y, Ryou JW. The dark side of CEO social capital: Evidence from real earnings management and future operating performance. Journal of Corporate Finance. 2021:101920. doi: https://doi.org/10.1016/j.jcorpfin.2021.101920

58.	Bhandari A, Mammadov B, Shelton A, Thevenot M. It is not only what you know, it is also who you know: CEO Network Connections and Financial Reporting Quality. Auditing: A Journal of Practice & Theory. 2018;37(2):27-50.

59.	Chahine S, Fang Y, Hasan I, Mazboudi M. CEO Network Centrality and the Likelihood of Financial Reporting Fraud. Abacus. 2021. doi: https://doi.org/10.1111/abac.12219

60.	Krishnan GV, Raman KK, Yang K, Yu W. CFO/CEO-board social ties, Sarbanes-Oxley, and earnings management. Accounting horizons. 2011;25(3):537-57.

61.	He Q, Li X, Zhu W. Political connection and the walking dead: Evidence from China's privately owned firms. International Review of Economics & Finance. 2018. doi: https://doi.org/10.1016/j.iref.2018.12.007

62.	Cao X, Pan X, Qian M, Tian GG. Political capital and CEO entrenchment: Evidence from CEO turnover in Chinese non-SOEs. Journal of Corporate Finance. 2017;42:1-14.

63.	Chaney PK, Faccio M, Parsley D. The quality of accounting information in politically connected firms. Journal of accounting and Economics. 2011;51(1-2):58-76.

64.	Bona-Sánchez C, Pérez-Alemán J, Santana-Martín DJ. Earnings credibility in politically connected family firms. The British Accounting Review. 2019;51(3):316-32.

65.	Tao Q, Sun Y, Zhu Y, Yang X. Political connections and government subsidies: Evidence from financially distressed firms in China. Emerging Markets Finance and Trade. 2017;53(8):1854-68.

66.	Hashmi MA, Brahmana RK, &, Lau E. Political connections, family firms and earnings quality. Management Research Review. 2018;41(4):414-32.

67.	Liu Y, Li X, Zeng H, & , An Y. Political connections, auditor choice and corporate accounting transparency: evidence from private sector firms in China. Accounting & Finance. 2017;57(4):1071-99.

68.	Bangladesh Securities and Exchange Commission. Annual Report 2018-2019. 2019. Available from: https://www.sec.gov.bd/home/annual_reports

69.	Huang XS, Sun L. Managerial ability and real earnings management. Advances in accounting. 2017;39:91-104.

70.	Kouki A. Mandatory IFRS adoption, investor protection and earnings management: A data analysis of Germany, France and Belgium listed companies. International Journal of Accounting & Information Management. 2018;26(1):187-204.

71.	Hope O-K, Thomas WB, Vyas D. Financial reporting quality of US private and public firms. The Accounting Review. 2013;88(5):1715-42.

72.	Dechow PM, Dichev ID. The quality of accruals and earnings: The role of accrual estimation errors. The accounting review. 2002;77(s-1):35-59.

73.	Kothari SP, Leone AJ, Wasley CE. Performance matched discretionary accrual measures. J Acc Econ. 2005;39(1):163-97.

74.	Abernethy MA, Kuang YF, Qin B. The influence of CEO power on compensation contract design. The Accounting Review. 2015;90(4):1265-306.

75.	Adams RB, Almeida H, Ferreira D. Powerful CEOs and Their Impact on Corporate Performance. The Review of Financial Studies. 2005;18(4):1403-32.

76.	Governance Innovation Unit. Advertisement of PM Fellowship 2020-21: Frequently Asked Questions view 2020 [updated October 5. Available from: http://www.giupmo.gov.bd/site/notices/8e40f4b2-c4c3-4e8d-8e8b-e66ae1a903f3/Advertisement-of-PM-Fellowship--2020-21-First-Phase--Cancelled---.

77.	Martin G, Campbell JT, Gomez-Mejia L. Family control, socioemotional wealth and earnings management in publicly traded firms. Journal of Business Ethics. 2016;133(3):453-69.

78.	Hsieh Y-T, Chen T-K, Tseng Y-J, Lin R-C. Top Management Team Characteristics and Accrual-Based Earnings Management. The International Journal of Accounting. 2018;53(4):314-34.

79.	Ullah S, Akhtar P, Zaefarian G. Dealing with endogeneity bias: The generalized method of moments (GMM) for panel data. Industrial Marketing Management. 2018;71:69-78.

80.	Roodman D. How to do xtabond2: An introduction to difference and system GMM in Stata. The stata journal. 2009;9(1):86-136.

81.	Shah SF, Rashid A, Malik WS. Potential Substitution Between Accrual Earnings Management and Real Earnings Management Among Pakistani Listed Firms. Global Business Review. 2020. doi: https://doi.org/10.1177/0972150920957617

82.	Uddin MA, Ali MH, Masih M. Political stability and growth: An application of dynamic GMM and quantile regression. Econ Modelling. 2017;64:610-25.

83.	Stubben SR. Discretionary revenues as a measure of earnings management. The Accounting Review. 2010;85(2):695-717.

84.	Roychowdhury S. Earnings management through real activities manipulation. Journal of Accounting and Economics. 2006; 42(3):335-70.

85.	Jensen MC. Self-interest, altruism, incentives, and agency theory. Journal of applied corporate finance. 1994;7(2):40-5.

86.	Ahmed S, Uddin S. Toward a political economy of corporate governance change and stability in family business groups: A morphogenetic approach. Accounting, Auditing & Accountability Journal. 2018. doi: https://doi.org/10.1108/AAAJ-01-2017-2833

87.	Sheikh S. An examination of the dimensions of CEO power and corporate social responsibility. Review of Accounting and Finance. 2019;18(2):221-44.

88.	Uddin S. Rationalities, domination and accounting control: A case study from a traditional society. Critical Perspectives on Accounting. 2009;20(6):782-94.

89.	Siddiqui J, Ferdous CS. The regulatory conundrum: Achieving corporate governance reforms in developing countries. Corporate governance in emerging markets: Springer; 2014. p. 445-64.
