# Supplementary material for: Do powerful CEOs matter for earnings quality? Evidence from Bangladesh
Source: PLoS One. 2023 Jan 20;18(1):e0276935. doi: 10.1371/journal.pone.0276935 (PMC9858436; doi:10.1371/journal.pone.0276935)
Supplement: Attachment — Submitted filename: Response to Reviewers (2).docx [file pone.0276935.s002.docx]

For all page numbers stated in this table, please use to the revised manuscript with track changes file.

| **Reviewer’s Comments** | **Author’s Response** |
| --- | --- |
| **Reviewer #1:** |  |
| **Some amendment need to be done in sections below:** |  |
| 1. Literature review- should include discussion on earnings quality | The following discussion about earnings quality is included at the beginning of literature review page 9&10:  Extant literature does not offer any single universally accepted definition of earnings quality. For example, Neel (27) mentioned that high quality earnings are those that are presented faithfully. In other words, quality earnings should be presented in a neutral, complete, and free from error condition (27). According to Dechow, Weili (28) quality of earnings depends on its’ decision usefulness. In other words, the higher the relevance of the earnings figure for any specific decision, the higher the quality of that earnings. Accordingly, Dechow, Weili (28) defined quality earnings as “higher quality earnings provide more information about the features of a firm’s financial performance that are relevant to a specific decision made by a specific decision-maker”. According to Dichev, Graham (29) high quality earnings are backed by actual cashflows, accurately reflect the results of the operation, and economic reality of the firm. They also suggested that consistent reporting choices over time, and the absence of one-time items improve level of earnings quality. This study assumes that high quality earnings are the results of a low level of earnings management practices (28). Earnings management occurs when managers use accounting choices or make operational decisions to hide the actual operating performance (30). In other words, high quality earnings should contain a lower level of abnormal accruals that result from the discretionary use of accounting rules (28) and should reflect a lower level of real activities management. Based on this concept of earnings quality, following Hope, Thomas (31) this study uses the accrual models to measure earnings quality. Dechow, Weili (28) suggest that the use of accrual models as a proxy for earnings quality has become an accepted method in accounting literature. |
| 2. Research approach- need to revise and revisit based on following items: |  |
| i. Explanation for excluding for firm less than 8 entities | Following Yamaguchi (2022), this study excludes firms in the industry consists of less than 8 entities to ensure sufficient data to measure the earnings quality proxies. The following explanation is added on page 19:  “*Next, we excluded the firms in the industry with less than 8 business entities to ensure sufficient data to measure the earnings quality proxies (61). Finally, this study excludes the firms with insufficient financial data and with insufficient financial data. The sample of this study comes from 8 industries. These are textile, engineering, food and allied, IT and telecom, fuel and power, cement and ceramics, pharmaceuticals, and miscellaneous.*” |
| ii. No of firms 165 for 10 years should be 1650 not 1395 | The following explanation is added on page 19:  *‘The panels of this study are not balanced.* *There are firms that have less than 10 years of data”.* |
| iii. Why 8 observations? Explain | This study following prior studies estimates the accrual models cross sectionally for each year and every industry with at least 8 observations to ensure sufficient data to capture the estimated parameters (Alzoubi, 2018; Oz & Yelkenci, 2018; Yamaguchi, 2022). The additional explanation is included on page 21:  *“This study following prior studies estimates the following regression cross sectionally for each year and every industry with at least 8 observations to ensure sufficient data to capture the estimated parameters (61-63)”* |
| iv. score of 1 if it is above or same as the sample median - confusing statement | The use of sample median as a benchmark to decide whether those elements generate power for a CEO has been used by prior studies (for instance Badolato et al. (2014, p. 226), Muttakin et al. (2018, p. 46), and Lisic et al. (2016, p. 33).This section has been revised to reflect better understanding of the scoring process used in this research (pages 21-22):  To measure CEO structural power, this study considers three elements - CEO’s board committees, CEO duality, and CEO only insider. First, this study counts the number of board committees on which the CEO has a role. When a CEO is on numerous board committees, it creates a concentration of decision-making in one person and may increase that individual’s power and ability to influence the board’s key decision (Abernethy et al., 2015). Eventually, the variable CEO’s board committees gets the score of 1 if a CEO’s presence in corporate board committees is equal or above the sample median, or 0 otherwise [74]. The use of sample median as a benchmark to decide whether the elements generate power for a CEO has been used by prior studies [37, 51, 65]. Next, CEO duality gets a score of 1 if board president and CEO is the same person, or 0 otherwise [31]. Finally, CEO only insider gets the score of 1 if CEO is the only person who represents top management in board meetings, or 0 otherwise [75]. This study then generates CEO structural power (*Strct_pwr*) by adding the score of CEO duality, CEO only insider, and CEO’s board committees. |
| v. CEO expert = experience years OR professional degree or BOTH | Yes, CEO expert is determine based on experience years or professional degree or both. The section has been revised to make the explanation clearer (page 22-23):  To measure CEO expert power, this study considers two elements: CEO tenure and CEO professional degree. This study considers the length of the CEO’s tenure as the first source of expert power because the ability to handle environmental uncertainties and to influence a firm’s key strategic decisions is likely to increase with greater tenure (51, 64). Second, a CEO with a chartered accountant or cost and management accountant or certified financial analyst degree indicates his or her accounting and financial expertise (19). This study assumes that if a CEO holds the post for a long time or achieves a professional accounting of finance degree, he or she can enjoy the privileges of expert power. CEO tenure is scored as 1 if the year of experience as a CEO is equal or greater than the sample median, or 0 otherwise. CEO professional degree is given a score of 1 if the CEO possesses a professional degree (CA or CMA or CFA), and 0 otherwise. Finally, the CEO is considered to have expert power (Expt_pwr) if the CEO has scored 1 for either the CEO tenure or CEO professional degree or both. |
| vi. Corporate board membership is it refer to CEO multiple directorships? | Yes |
| vii. . Improve the measurement of model 1 variables | The write up about the measurement of model 1 variables has been improved (page 28 & 29): ${EQ}_{it}$ \| = \| denotes earnings quality proxies (DisTA, DisTAC, DisREV, and Ab_P_C); ${EQ}_{it-1}$ \| = \| means prior year value of the respective earnings quality proxy; *Strct_pwr* \| = \| structural power (CEO duality + only insider + presence in board committees); *Own_pwr* \| = \| ownership power (1 if the percentage of a CEO’s stock ownership or the percentage of a CEO’s family members' ownership is greater than or equal to the sample median, or 0 otherwise).; *Expt_pwr* \| = \| expert power (1 if the year of experience as a CEO is greater than or equal to the sample median or if a CEO possesses a professional degree (CA or CMA or CFA), or 0 otherwise).; *Prtge_pwr* \| = \| prestige power (CEO’s other corporate board directorship + non-profit board directorship + elite education); *Polit_pwr* \| = \| political power (1 if CEO is a minister or member of parliament or government official or close relative to a politician or top post holder in the ruling party, or 0 otherwise).; *Firm_siz* \| = \| total assets of a firm (in natural log form); *Levge* \| = \| proportion of total debt to total assets; *RoA* \| = \| proportion of net operating income to average assets; *AgE* \| = \| total years since the company is founded (in natural log form); *NegEarn* \| = \| 1 if the firm records a net loss in a year, or 0 else; *Big 4* \| = \| 1 if a firm’s financial reports are attested by a Big 4 audit firm, or 0 else; *Growth_Rev* \| = \| percentage change in revenues from the immediate past year; *B_size* \| = \| board size (number of directors on the corporate board of directors); *B_Ind* \| = \| board independence (percentage of independent directors on the corporate board of directors; *Inst_Own* \| = \| institutional ownership (percentage of a firm’s share held by institutional investors; $\eta_{t}$ \| = \| time-specific effect; $\varphi_{i}$ \| = \| industry-specific effect; $\upsilon_{it}$ \| = \| the error term |
| 3. Results- check for the result- figure in table should be consistent with text. |  |
| i. suggest to provide the descriptive table based on the raw data before coded it to 0,1 till 8. | Descriptive statistics of the elements of power dimensions are included in the manuscript (pages 32-33). |
| ii. Suggest to place a legend for each of variables mentioned in Table 2 | Legend for each of variables mentioned in Table 2 is included (page 31 & 32). |
| iii. Suggest may also include DV in correlation table as to determine the strength of relationship between DV and IVs | DV is added in the correlation matrix. Please see page 34. |
| iv. May provide an explanation/ discussion/ justification of result for relationship between CEO power & EQ not only mention the statistical results. | Justifications are added in the manuscript. Please see pages 35- 38.  **Structural power:**  These results also imply that structurally powerful CEOs in Bangladesh manage accruals to show better firm performance. These results are consistent with prior studies by Koo and Kim (2019), Mande and Son (2012), Nuanpradit (2019), Bouaziz et al. (2020), Shiah-Hou (2021) and Muttakin et al. (2017). These results are consistent with the arguments by prominent agency theorists that excessive formal power promotes CEO entrenchment, which in turn results in actions that deviate from the interests of residual claimants, and ultimately they lose their wealth (Fama & Jensen, 1983; Finkelstein & D'aveni, 1994; Jensen & Meckling, 1976). Ahmed and Uddin (2018) mentioned that CEOs in Bangladesh have enormous power, and other top executives are prone to follw the instructions of CEOs even at the expense of general shareholders. This is because the recruitments, promotions, bonuses and fireing from the job are not performed in a formal way. These tasks are performed informally, arbitarily, and with direct intervesions by CEOs (Ahmed & Uddin, 2018).  **Ownership power:**  These results are consistent with Le et al. (2020), Hribar and Nichols (2007) and Rashid (2016). These results suggest that CEOs with high ownership power are not associated with the decrease in earnings quality. There could be at least three explanations for these findings. First, Some scholars argue that a CEO may hold different dynamics of power, but it is not necessary to use all the bases of power to achieve any objective (Kovach, 2020; Robbins & Judge, 2017). Second, unlike CEOs with little or no equity ownership, the compensation and employment contracts of CEOs with high ownership power are not sensitive to the firm’s accounting performance (Meo et al., 2017). As a result, they do not need to mask poor financial performance through earnings management techniques (Meo et al., 2017). Third, the identity and goodwill of the CEOs with high ownership stakes are associated with their firms (Berrone et al., 2012). Thus, they have the fear of losing their reputation if they engage in any accounting manipulation.  **Expert power:**  These results are logical in the context of Bangladesh, as in many cases, expert CEOs both manage and control corporate decisions and take actions that debilitate the wealth of minority shareholders (Ahmed & Uddin, 2018). The results are also in line with agency theory propositions that managerial expertise augments managerial power, which leads managers to engage in more earnings management practices to prevent information leaks about their entrenched behaviors (Chihua et al., 2016; Ding et al., 2007; Finkelstein, 1992).  **Prestige power:**  One possible reason for the insignificant effect is that CEOs with high prestige power do not engage in unethical practices to protect their reputation and to increase their social status.  **Political power:**  These results are logical for at least two reasons. First, although the politically connected CEOs make a huge amount of profit, they do not care about depriving non-controlling shareholders of their financial rights by manipulating financial statements. They transfer the resources of a cash cow firm to their different private projects and declare and pay very insignificant dividends (Uddin, 2009).  Second**,** regulatory bodies play a silent role regarding the wrongdoing of politically connected CEOs. This is because if they take any action against these politically connected CEOs, they may even lose their position or may be shifted to other less important sections of the government (Ahmed & Uddin, 2018). Interestingly, the top positions of corporate regulatory bodies are also filled by explicitly politically affiliated people with the ruling party. As a result, these top officials of regulatory bodies offer preferential treatment to politically connected CEOs as they follow the same political ideology. |
| v. Need to justify as CEO ownership is on average 77% but the result is insignificant | Justifications are included in the manuscript on page 36.  There could be at least three explanations for these findings. First, Some scholars argue that a CEO may hold different dynamics of power, but it is not necessary to use all the bases of power to achieve any objective (Kovach, 2020; Robbins & Judge, 2017). Second, unlike CEOs with little or no equity ownership, the compensation and employment contracts of CEOs with high ownership power are not sensitive to the firm’s accounting performance (Meo et al., 2017). As a result, they do not need to mask poor financial performance through earnings management techniques (Meo et al., 2017). Third, the identity and goodwill of the CEOs with high ownership stakes are associated with their firms (Berrone et al., 2012). Thus, they have the fear of losing their reputation if they engage in any accounting manipulation. |
| vi. Suggest to exclude predicted sign for control variables in Table 4, 5 & 6 | Predicted signs are removed. |
| vii. Additional analysis-   Justify why only production cost as Roychowdhury (2016) focused on 3 elements of REM which is CFO, production & discretionary expenses. | Justifications are included in the manuscript on page 45:  Although Roychowdhury (2006) mentioned three distinctive ways of real activity management: cash flow manipulation; discretionary expense manipulation; and production cost manipulation, the production cost manipulation system is mainly used by manufacturing firms (Alhadab & Clacher, 2018). This study tests only the production cost manipulation for additional analysis purposes, as 88% of our sample firms are manufacturing firms. Thus, the probability is higher that the CEOs will prefer production cost manipulation to discretionary expense manipulation or cash flow manipulation for the purpose of real earnings management. |
| viii. Table 6- Explain why only 161 firms not 165. | The statistical software STATA deleted the data of four firms due to some missing observations. But this study included the data of all 165 firms before running the regressions. |
| 4. Discussion |  |
| Further, the lack of skilled, competent, and independent manpower in regulatory bodies is also a prime cause for the poor monitoring system- A statement based on previous study- any reference? it is not tested in this study. | *This part is excluded. We revised the conclusion section on pages 48- 49:*  *This study contributes to the literature by providing empirical evidence regarding the effect of a CEO's structural, ownership, expert, prestige, and political power on earnings quality in an emerging economy context. Using accrual earnings quality proxies, real earnings management measures, and two-step system GMM, our results show that CEOs with greater structural power, considerable expertise, and politically connected CEOs are involved in inferior financial reporting practices. These CEOs, besides accrual earnings management, apply real activity management techniques to manipulate the reported earnings. However, CEOs with high ownership and prestige power have an insignificant effect on earnings quality. These results suggest that powerful CEOs, especially those with excessive structural and expert power and politically connected CEOs, are detrimental to the interests of residual claimants (specifically minority stockholders) in emerging economies like Bangladesh.*  *The results of this study have some crucial policy suggestions. This study offers mainly two policies. First, a restriction on CEOs’ direct political affiliation may improve the corporate regulatory environment and, ultimately, the reported earnings quality. This will reduce CEOs’ undue pressure over regulatory bodies. Second, a limit on CEOs’ tenure and the involvement of one or two top executives other than the CEO on the corporate board may significantly improve internal corporate governance. Their consequent involvement in strategic decision-making will minimize CEOs’ autocracy.*  *This study has two main limitations. First, for earnings quality measures, this study used only accounting-based accrual earnings quality proxies and one real earnings management proxy. Second, as this study did not find appropriate external instruments, this study used only internal instruments to address the issues of endogeneity. Future studies may test the effects of different dimensions of CEO power on cash flow manipulation, discretionary expense manipulation, and the market-based earnings quality measures. Future research may also investigate whether the effects of powerful CEOs on earnings quality differ between family-controlled and non-family firms. Finally, future research may focus on using external exogenous instruments to address the endogeneity in CEO power dimensions.* |
| **Reviewer #2:** | No comment stated |
| **Reviewer #3:** |  |
| 1. Authors should divide the sample on the basis of firm size (small firms and large firms) to know the how CEO’s Power affect firm earning quality for small firms and large firms as small firms are different than large firms. Authors should also cite the following studies while discussing about financing constraints in terms of firm size. | Based on the reviewer's comment, the authors divided the sample into two groups (large and small firms), taking the average firm size as the cutoff point for dividing the sample. Then we run the regression. The regression results are shown in Appendix 1. As can be seen from the table in Appendix 1, structural, expert, and political power have a negative effect on earnings quality in both large and small firms. Except for structural power in large firms (significant at a 10% level), the effect of these variables on large and small firms is statistically insignificant. Also, the effect of ownership power on earnings quality is positive for both large and small firms and statistically significant only for large firms (at a 5% level). Finally, prestige power has an insignificant effect on both large and small firms. Thus, the results show an insignificant difference between the two sets of samples. Eventually, the assumption that, as small firms have resource constraints, they will be involved in more earnings management than larger firms is not applicable for the samples in this study. The authors argue that the capital market of Bangladesh is small and the difference among the listed non-financial firms in terms of firm size or total assets is insignificant. As can be seen from the descriptive statistics, the standard deviation of firm size is very low at 1.52. Also, the mean, median, and third quartile values are 21.67, 21.65, and 22.58, respectively, which are very close to each other. Thus, the insignificant difference between large and small firms in terms of the powerful CEO effect on earnings quality is logical. For the insignificant difference, the authors excluded the additional analysis. |
| Authors should also cite the following studies while discussing about financing constraints in terms of firm size.   a. Gertler, M. and Gilchrist, S. (1993), “The role of credit market imperfections in the monetary transmission mechanism: arguments and evidence”, The Scandinavian Journal of Economics, Vol. 95 No. 1, pp. 43-64. | The referred paper is not cited as the additional analysis is not included in the manuscript. |
| b. Gertler, M. and Gilchrist, S. (1994), “Monetary policy, business cycles, and the behavior of small manufacturing firms”, Quarterly Journal of Economics, Vol. 109 No. 2, pp. 309-340. | The referred paper is not cited as the additional analysis is not included in the manuscript. |
| Gupta, G., Mahakud, J. and Verma, V. (2021), "CEO's education and investment–cash flow sensitivity: an empirical investigation", International Journal of Managerial Finance, Vol. 17 No. 4, pp. 589-618. | The referred paper is not cited as the additional analysis is not included in the manuscript. Also, the dependent variable of the referred paper is different from ours. Finally, we focused only on CEO Professional Degree (CA, CMA, CFA), and do not consider bachelor or master’s degree. |
| 2. Authors need to write additional hypothesis for small and large size firms analysis. | As the additional analysis is not included, the addition hypothesis for small and large size firms’ analysis is not required. |
| 3. Results of the empirical analysis are clearly presented. But after the implemented changes mentioned for the financial constraints, it should be re-written accordingly. | Results are not affected as the additional analysis is not included. |
| 4. Authors should also include the following studies of CEO’s characteristics to strengthen their literature review. 1. Gupta, G., 2022. CEO's age and investment‐cash flow sensitivity. Managerial and Decision Economics. | The referred paper is not cited as the IV and DV are different from ours. Our study does not include any of the variables, CEO age and the investment‐cash flow sensitivity. |
| 2. Farag, H., & Mallin, C. (2018). The influence of CEO demographic characteristics on corporate risk-taking: Evidence from Chinese IPOs. The European Journal of Finance, 24, 1528–1551 | Cited on page 16. The sentence that contains the reference is inserted here:  *Further, prestige power induces CEOs to take risky initiatives since their social ties help to get new ideas, protect jobs, and improve the chances of re-employment after the CEO is fired for failed risky strategies (Dbouk et al., 2020; Farag & Mallin, 2018; Griffin et al., 2021).* |
| 3. Gupta, G. (2021). CEO's educational background, economic policy uncertainty and investment-cash flow sensitivity: Evidence from India. Applied Economics, 1–12. | Not cited as the dependent, independent, and the moderating variables do not match with our study. |
| 4. Naheed, R., Jawad, M., Naz, M., Sarwar, B., & Naheed, R. (2021). Managerial ability and investment decisions: Evidence from Chinese market. Managerial and Decision Economics, 42, 985–997. | Not cited as the dependent variable does not match with our study. |
| **Reviewer #4:** |  |
| Major issues:   1. You need to elaborate on research gaps further and then link those gaps to the contributions in results which will help you motivate the research context. | The write up about research gaps is extended in the introduction section on pages 2-3. The additional write-up is inserted here:  A detailed review of existing literature closely associated with this study reveals that 64% of the prior studies (Ali & Zhang, 2015; Baker et al., 2019; Fabrizi & Parbonetti, 2017; Francis et al., 2008; Gounopoulos & Pham, 2018; Harris et al., 2019; Hsieh et al., 2014; Khanna et al., 2015; Koo & Kim, 2019; Leon & ChiiShyan, 2017; Mande & Son, 2012; Meo et al., 2017; Petrou & Procopiou, 2016; Shiah-Hou, 2021) (14 out of 22) focused on a developed economy, the US (United States). Whereas only 32% of prior studies (Chihua et al., 2016; Hsieh et al., 2018; Hu & Gan, 2017; Le et al., 2020; Nuanpradit, 2019; Sani et al., 2020; Wang et al., 2017) (7 out of 22) focused on emerging economies namely China, Taiwan, Thailand, Vietnam, and Nigeria. One study focused on the French context (Chikh & Filbien, 2011). Thus, there is a lack of empirical evidence regarding the effect of powerful CEOs on earnings quality from emerging nations.  A deeper look reveals that studies that focused on the US economy provide mixed evidence. The results of 71% of studies (Ali & Zhang, 2015; Baker et al., 2019; Francis et al., 2008; Harris et al., 2019; Hsieh et al., 2014; Khanna et al., 2015; Koo & Kim, 2019; Leon & ChiiShyan, 2017; Mande & Son, 2012; Shiah-Hou, 2021) (10 out of 14 ) suggest that powerful CEOs are detrimental to earnings quality, whereas 29% of studies (Fabrizi & Parbonetti, 2017; Gounopoulos & Pham, 2018; Meo et al., 2017; Petrou & Procopiou, 2016) (4 out of 14) suggest that powerful CEOs are associated with improved earnings quality. The inconsistency in empirical findings is much stronger in emerging economies. The empirical results of 43% of studies (Hu & Gan, 2017; Le et al., 2020; Nuanpradit, 2019) (3 out of 7) that focused on emerging nations suggest that powerful CEOs are associated with more earnings management. Whereas 57% of studies (Chihua et al., 2016; Hsieh et al., 2018; Sani et al., 2020; Wang et al., 2017) (4 out of 7) suggest that powerful CEOs are beneficial for reported earnings quality. Thus, the extant literature provides inconsistent findings regarding the effect of powerful CEOs on earnings quality. This inconsistency in findings suggests that there is merit in investigating this relationship. |
| Literature review and hypotheses Development - please enhance your hypotheses by: (i) drawing on the theory; (ii) empirical literature; (iii) research setting/contextual insights; and (iv) then setting up your hypotheses. You will do this for each hypothesis. You can do so by drawing on both seminal (old) and recently (newly) published studies. | The write-up of the literature review for each of the power dimensions is enhanced by following the most recent and seminal papers and drawing on papers that are based on the Bangladesh context. Please see pages 11- 18. The additions are inserted here.  **Structural power and earnings quality:**  Excessive concentration of structural power, which is based on formal organizational structure and hierarchical authority for CEOs, facilitates self-enhancement attempts among CEOs with self-serving motives (Blagoeva et al., 2020). This in turn reduces reported earnings quality. Scholars argue that the position of a CEO itself is a source of structural power (Sheikh, 2019). Furthermore, the addition of other elements of dominance, such as being the chair of the board, being the only top management representative on the board, and being a member of different board committees, adds stronger layers to the structural power of a CEO. This high level of structural power opens the door for the CEO to create undue pressure over other top executives for earnings management to inflate earnings (Florackis & Sainani, 2021).  Muttakin et al. (2017) in the context of Bangladesh documented a significant positive association between CEO duality and accrual earnings management.  In the context of Bangladesh, CEOs with high structural power enjoy unrestricted decision-making power and have complete access to all inside information (Ahmed & Uddin, 2018). Muttakin et al. (2018) argued that CEOs in Bangladesh are mainly family CEO, and they are very much entrenched with less concern about legitimacy and accountability. Other top executives enjoy very little formal power within the organization compared to CEOs. Normally, they do not have the membership of the board of directors. Joseph et al. (2014) argued that the situation of CEO only insider to board offers CEOs an information brokerage position and remove potential internal challengers.  **Ownership power and earnings quality:**  Further, several studies found insignificant effect. For example, using samples from listed Vietnamese firms, Le et al. (2020) identified an insignificant association between CEO ownership power and earnings management. Also, Hribar and Nichols (2007) found an insignificant effect of managerial equity incentives on earnings management after controlling the standard deviation of sales and cashflows. Finally Rashid (2016) in the context of Bangladesh identified a statistically insignificant association between managerial ownership and agency costs. Thus, whether CEOs with high ownership power are associated with improved earnings quality or decrease the quality of earnings is an unsettled question that demands further investigation.  **Expert power and earnings quality:**  Following Finkelstein [29], this study focuses on CEOs’ functional expertise since functional expertise also opens the door to enjoying decision-making autonomy and practising managerial autocracy (Altunbaş et al., 2018; Combs et al., 2007).  Earlier studies mentioned that managers with longer tenure and more knowledge gain greater power over a firm's operating strategies, which induces them to be involved in more earnings manipulation activities (Finkelstein, 1992; Hsieh et al., 2018). Long tenure also makes it easier for CEOs to establish a good relationship with other top executives. As other top executives are also stimulated by personal financial gain, it becomes easy for CEOs to implement strategies that fit their own preferences as their tenure advances (Darouichi et al., 2021; Strebulaev & Yang, 2013). Further, long tenure helps CEOs establish control over internal monitoring mechanisms (Darouichi et al., 2021; Wen et al., 2002). In Bangladesh, there are instances where the CEO himself becomes part of the monitoring mechanism by being a member of the audit committee. Thus, in these cases, the monitoring organ of a firm plays a ceremonial role. This paves the way to engaging in self-serving behaviours detrimental to minority shareholders.  Besides tenure, CEOs with professional accounting or finance qualifications are very much familiar with the firms’ financial reporting process and favorable accounting treatments (Salehi et al., 2018). Therefore, they can strategically employ different earnings management devices to convey a preferred financial report (Ferramosca & Allegrini, 2018; Rashid, 2016). Prior studies considering the Bangladesh context documented that firms’ accounts departments are acquiescent with the wishes and decisions of powerful chief executives and they instruct based on their interests and desires (Ahmed & Uddin, 2018; Uddin, 2009). Uddin (2009), referring to the Bangladesh context, documented that CEOs play the key role in preparing the sales budget and production managers remain ready to execute his or her instructions. The decision of the CEO is final regarding any production issue. This situation also creates an ample opportunity for CEOs to get involved in real earnings management through abnormal production costs.  In their most recent study, Altarawneh et al. (2022) find that long-tenured CEOs are associated with improved financial reporting quality.  **Prestige power and earnings quality:**  Finkelstein (1992) argues that managerial prestige is based on a person’s reputation in institutional environments, social organizations, and among stakeholders. Also, managers’ elite educational backgrounds are an important source of prestige (Finkelstein, 1992; Lisic et al., 2016). Attending elite educational institutions paves the way to familiarizing themselves with other business elites and creating useful networks of expertise (Tholen et al., 2013).  Further, prestige power induces CEOs to take risky initiatives since their social ties help to get new ideas, protect jobs, and improve the chances of re-employment after the CEO is fired for failed risky strategies (Dbouk et al., 2020; Farag & Mallin, 2018; Griffin et al., 2021).  We expect that as CEOs with high-prestige power sit on numerous corporate boards and maintain strong social networks, it is easy for them to manipulate revenues and expenses if required. Fang et al. (2022) argue that the reciprocal relationship of CEOs with other corporate boards helps them to initiate earnings management through timing sales and expenses, utilizing relationship sales, or introducing asset exchange transactions.  A few studies evidence that CEOs with greater connections maintain a high quality of accrual earnings (Altarawneh et al., 2022; Bhandari et al., 2018), and these CEOs are associated with minimal fraudulent incidents (Chahine et al., 2021). However, some other studies document a positive association between well-connected CEOs and earnings management (Fang et al., 2022; Griffin et al., 2021; Krishnan et al., 2011).  **Political power and earnings quality:**  In the context of Bangladesh, politically connected CEOs need not care about rules and regulations. Rules and regulations are for managers with low power. Ahmed and Uddin (2018) mentioned that politically connected business leaders in Bangladesh are often able to ignore or avoid rational initiatives taken by state regulatory bodies. The extent of following corporate governance codes or guidelines is up to their comfort level (Ahmed & Uddin, 2018). If they feel any prospective negative outcome for themselves by following a particular instruction from the regulatory body, they simply ignore it or even mention that they executed the instruction without actually following it (Ahmed & Uddin, 2018). Sobhan (2016) in the Bangladesh context documented a significant overstatement of compliance with corporate governance guidelines in annual reports. |
| Methodology- You need to add other corporate governance variables as control variables such as board size, board independence and ownership structure. | Three corporate governance variables: board size, board independence and institutional ownership are included as control variable. This study estimated the adjusted econometric model again and revised all the results accordingly. The adjusted econometric model can be seen on page 28 and the revised results can be seen in Table 4 (pages 40–41), Table 5 (pages 43–44), and Table 6 (pages 47–48). |
| 1. You need to explain the GMM approach further and show which instruments you use as exogenous variable(s) and why. If you did not consider any instrumental variable(s), you should use 2SLS to make sure that your results are not driven by endogeneity issues. In your descriptive, I can notice that you may use propensity score matching to sole self-selection bias. | GMM is explained in detail in the manuscript on pages 26- 27. The additional explanations are inserted here:  As the panel data for his study is dynamic, this study estimates the relationship between CEO power and earnings quality using the two-step system generalized method of moments (GMM), developed by Blundell and Bond (1998). Prior studies mentioned that the dynamic panel GMM estimator yields consistent outcomes in the existence of dynamic endogeneity, heterogeneity, and simultaneity (Ullah et al., 2018; Wintoki et al., 2012). As in dynamic panel data, the cause-and-effect relationship is generally dynamic over time. In GMM, lags of the dependent variables are used as explanatory variables to control the dynamic panel bias, also called dynamic endogeneity (Nickell, 1981; Roodman, 2009; Ullah et al., 2018). So, the lag-dependent variables work as an instrument to control the endogenous relationship. In GMM, the endogeneity of regressors is removed by internally transforming the variables. There are two types of transformation: first difference transformation, or one-step GMM, and second order transformation, or two-step GMM. In the first difference transformation, the past value of a variable is subtracted from its present value (Roodman, 2009; Ullah et al., 2018). But the problem with this transformation method is the chance of data loss if the panels are unbalanced and there are missing values (Arellano & Bover, 1995; Duru et al., 2016; Ullah et al., 2018). To solve the problem, Arellano and Bover (1995) introduced the forward orthogonal deviation method, in which the mean of future available values of a variable is subtracted from the current value. Arellano and Bover (1995) proved that this transformation removes the unobserved fixed effects and at the same time preserves the orthogonality among the transformed errors.  This study used the xtabond2 codes written for statistical software STATA by Roodman (2009) to get system GMM estimators for our unbalanced panels. In all the estimations, Windmeijer (2005) finite-sample correction to the standard errors is used to get more precise estimates. Scholars suggest that without this correction, reported standard errors will be severely biased (Roodman, 2009; Windmeijer, 2005). This study used a one-period lag of earnings quality proxies to complete the dynamic process, as lags beyond one period show an insignificant coefficient. This study used only internally generated instruments that are lags of instrumented variables.  Consistent with prior studies, this study used two post estimations to test the appropriateness of the econometric model used: the Arellano-Bond test and the Hansen *J* test (Ullah et al., 2018; Wintoki et al., 2012). One of the prime requirements for the validity of GMM results is that the instruments used are exogeneous. The Hansen *J* test is used to determine whether the instruments are exogeneous or not under the null hypothesis that the instruments are exogeneous (Wintoki et al., 2012). An insignificant test result suggests that instruments are correctly specified. In addition to that, another important requirement for the validity of GMM results is that there is no serial correlation in the idiosyncratic disturbance term (Arellano & Bond, 1991; Ullah et al., 2018; Wintoki et al., 2012). Alternatively, it means the lagged variables are uncorrelated with the random error term. Wintoki et al. (2012) suggest that if this assumption remains valid, then the residuals in the first difference (AR (1)) should be correlated. However, the residuals in the second differences (AR (2)) should be uncorrelated. The test results of the Arellano-Bond test and Hansen *J* statistics are presented after each estimation for this study. All continuous variables have been winsorized at the 1% and 99% level to neutralize the effect of outliers in our data set (Anagnostopoulou & Tsekrekos, 2017; Bruynseels & Cardinaels, 2014; Choi et al., 2021).This study used internally generated instruments that are lag or past values and past changes of the endogenously determined regressors (such as structural power, ownership power, expert power, political power, and firm size) as exogenous instruments. The exogeneity of used instruments has been proved by the Hansen J test. The use of the internally generated instruments removes the need to find appropriate external instruments and the use of 2SLS to solve the endogeneity issues. |
| 2. In your descriptive, I can notice that you may use propensity score matching to sole self-selection bias. | The self-selection-based endogeneity may arise mainly from two sources with respect to this study. First, if samples suffers from self-selection or samples are not representative of the population (Antonakis et al., 2010; Hill et al., 2021). As can be seen from Table 1, sample selection procedure (on page 20 of this manuscript), the population for this study, non-financial firms, consists of 215 firms (317–102). The final sample for this study consists of 165 firms, which represents 77% (165/215) of the entire population. Thus, it can be argued that the samples of this study properly represent the population and significantly minimize the chance of bias that results from the selection into samples. The second source of selection bias is the omitted bias, which results from not including other important variables that may affect the dependent variable (Ullah et al., 2018; Zaefarian et al., 2017). As can be seen from the estimation model (page 20) of this study, the model now includes 12 control variables, including year and industry dummies (board size, board independence, and institutional ownership are added based on the reviewer’s comment) that may affect the earnings quality. Thus, the chance of omitted bias has been significantly reduced. Finally, this study used the GMM (Generalized Method of Moments) estimation technique for this study. Zaefarian et al. (2017, p. 42) suggest that the GMM can control all the sources of endogeneity for dynamic panel data. Eventually, the authors argue that the coefficients of the CEO power dynamics are not affected by the self-selection bias. |
| 3. You use earnings management proxies to test earnings quality, why? Why you did not use the earnings response coefficient! | Dechow et al. (2010) in their review paper listed a wide range of earnings quality proxies used by prior studies. This study argues that the selection of earnings quality proxy is largely dependent on how one perceives earnings quality. This study assumes that high quality earnings are the results of a low level of earnings management practices (Dechow et al., 2010). Earnings management occurs when managers use accounting choices or make operational decisions to hide the actual operating performance (Healy & Wahlen, 1999). In other words, high quality earnings should contain a lower level of abnormal accruals that result from the discretionary use of accounting rules (Dechow et al., 2010), and should reflect a lower level of real activities management. Based on this concept of earnings quality, following Hope et al. (2013) this study uses the accrual models to measure earnings quality. Dechow et al. (2010) suggest that the use of accrual models as a proxy for earnings quality has become an accepted method in accounting literature.  However, the market-based earnings quality proxy, earnings response coefficient (ERC), assumes that the quality of earnings is solely dependent on the decision usefulness of earnings in equity valuation by investors (Dechow et al., 2010). In other words, investors’ responsiveness to earnings information is treated as earnings quality, and proxied by ERC. As this definition is out of the scope of this study, this study did not include the ERC as a proxy for this study. Further, the objective of this study is to detect intentional earnings management practices that reduce the decisional usefulness of earnings. But there are evidences that ERC cannot capture intentional earnings management practices (Dechow et al., 2010). Accordingly, Dechow et al. (2010) suggest that the use of ERC as a proxy of earnings quality is questionable if author or authors perceive high earnings quality that represents lower earnings management. Finally, in the context of an emerging economy that is characterized by weak regulatory environment, managers usually use earnings management practices to inflate earnings with a view to increase market responsiveness. So, if we use ERC as a proxy for earnings quality, we need to change our existing view of earnings quality. |
| Empirical findings - please link your findings more strongly to the: (i) theory, (ii) empirics, (iii) context; and (iv) highlight their economic, academic/research and policy implications. Closely link up and cite the papers that you have discussed in the background, theory and empirical literature review & and hypotheses development section to the findings you are presenting here | Justifications of the findings based on the context of Bangladesh are included. These results are linked with theoretical propositions and the papers that were cited in the literature review. Please see pages 35 – 38. The additional write-up is pasted here.  **Justifications for H1 findings :**  These results also imply that structurally powerful CEOs in Bangladesh manage accruals to show better firm performance. These results are consistent with prior studies by Koo and Kim (2019), Mande and Son (2012), Nuanpradit (2019), Bouaziz et al. (2020), Shiah-Hou (2021) and Muttakin et al. (2017).  These results are consistent with the arguments by prominent agency theorists that excessive formal power promotes CEO entrenchment, which in turn results in actions that deviate from the interests of residual claimants, and ultimately they lose their wealth (Fama & Jensen, 1983; Finkelstein & D'aveni, 1994; Jensen & Meckling, 1976). Ahmed and Uddin (2018) mentioned that CEOs in Bangladesh have enormous power, and other top executives are prone to follw the instructions of CEOs even at the expense of general shareholders. This is because the recruitments, promotions, bonuses and fireing from the job are not performed in a formal way. These tasks are performed informally, arbitarily, and with direct intervesions by CEOs (Ahmed & Uddin, 2018).  **Justifications for H2 findings:**  These results are consistent with Le et al. (2020), Hribar and Nichols (2007) and Rashid (2016). These results suggest that CEOs with high ownership power are not associated with the decrease in earnings quality. There could be at least three explanations for these findings. First, Some scholars argue that a CEO may hold different dynamics of power, but it is not necessary to use all the bases of power to achieve any objective (Kovach, 2020; Robbins & Judge, 2017). Second, unlike CEOs with little or no equity ownership, the compensation and employment contracts of CEOs with high ownership power are not sensitive to the firm’s accounting performance (Meo et al., 2017). As a result, they do not need to mask poor financial performance through earnings management techniques (Meo et al., 2017). Third, the identity and goodwill of the CEOs with high ownership stakes are associated with their firms (Berrone et al., 2012). Thus, they have the fear of losing their reputation if they engage in any accounting manipulation.  **Justifications for H3 findings:**  The findings of this study are consistent with Shiah-Hou (2021), Hu and Gan (2017), Le et al. (2020), Chihua et al. (2016), Altunbaş et al. (2018), and Priscilla and Siregar (2020). These results are logical in the context of Bangladesh, as in many cases, expert CEOs both manage and control corporate decisions and take actions that debilitate the wealth of minority shareholders (Ahmed & Uddin, 2018). The results are also in line with agency theory propositions that managerial expertise augments managerial power, which leads managers to engage in more earnings management practices to prevent information leaks about their entrenched behaviors (Chihua et al., 2016; Ding et al., 2007; Finkelstein, 1992).  **Justifications for H4 findings:**  One possible reason for the insignificant effect is that CEOs with high prestige power do not engage in unethical practices to protect their reputation and to increase their social status.  **Justifications for H5 findings:**  These results are logical for at least two reasons. First, although the politically connected CEOs make a huge amount of profit, they do not care about depriving non-controlling shareholders of their financial rights by manipulating financial statements. They transfer the resources of a cash cow firm to their different private projects and declare and pay very insignificant dividends (Uddin, 2009).  Second**,** regulatory bodies play a silent role regarding the wrongdoing of politically connected CEOs. This is because if they take any action against these politically connected CEOs, they may even lose their position or may be shifted to other less important sections of the government (Ahmed & Uddin, 2018). Interestingly, the top positions of corporate regulatory bodies are also filled by explicitly politically affiliated people with the ruling party. As a result, these top officials of regulatory bodies offer preferential treatment to politically connected CEOs as they follow the same political ideology. |
| Conclusion - Please outline a summary of findings, contributions, implications, limitations and avenues for future research. Especially, expand the discussions relating to implications, limitations and avenues for future research. | Conclusion is revised based on the above suggestions. Limitations and scope for future research are included on pages 48 – 49. The revised write-up following the instructions is inserted here.  This study contributes to the literature by providing empirical evidence regarding the effect of a CEO's structural, ownership, expert, prestige, and political power on earnings quality in an emerging economy context. Using accrual earnings quality proxies, real earnings management measures, and two-step system GMM, our results show that CEOs with greater structural power, considerable expertise, and politically connected CEOs are involved in inferior financial reporting practices. These CEOs, besides accrual earnings management, apply real activity management techniques to manipulate the reported earnings. However, CEOs with high ownership and prestige power have an insignificant effect on earnings quality. These results suggest that powerful CEOs, especially those with excessive structural and expert power and politically connected CEOs, are detrimental to the interests of residual claimants (specifically minority stockholders) in emerging economies like Bangladesh.  The results of this study have some crucial policy suggestions. This study offers mainly two policies. First, a restriction on CEOs’ direct political affiliation may improve the corporate regulatory environment and, ultimately, the reported earnings quality. This will reduce CEOs’ undue pressure over regulatory bodies. Second, a limit on CEOs’ tenure and the involvement of one or two top executives other than the CEO on the corporate board may significantly improve internal corporate governance. Their consequent involvement in strategic decision-making will minimize CEOs’ autocracy.  This study has two main limitations. First, for earnings quality measures, this study used only accounting-based accrual earnings quality proxies and one real earnings management proxy. Second, as this study did not find appropriate external instruments, this study used only internal instruments to address the issues of endogeneity. Future studies may test the effects of different dimensions of CEO power on cash flow manipulation, discretionary expense manipulation, and the market-based earnings quality measures. Future research may also investigate whether the effects of powerful CEOs on earnings quality differ between family-controlled and non-family firms. Finally, future research may focus on using external exogenous instruments to address the endogeneity in CEO power dimensions. |
| Minor issues  The standard of writing needs some work. A more careful reading and restructuring will help eliminate some remaining typos. Please refer to some relevant literature from the journal and wider literature such as the below references. | A professional proofreading service for this manuscript has been taken. This manuscript added 47 new references to improve its quality based on the reviewers’ comments. This manuscript now contains a total of 136 references. Previously it was 89 references. Please see the manuscript file. |

**Appendix 1: Association between CEO power dynamics and earnings quality with respect to large and small firms**

|  | **Large Firms** | **Small Firms** |
| --- | --- | --- |
|  | DisTAC | DisTAC |
| L. DisTAC | 0.161^*^ | 0.264^***^ |
|  | (0.094) | (0.001) |
| Strct_pwr | -0.062^*^ | -0.019 |
|  | (0.060) | (0.297) |
| Own_pwr | 0.057^**^ | 0.007 |
|  | (0.042) | (0.822) |
| Expt_pwr | -0.041 | -0.023 |
|  | (0.176) | (0.241) |
| Prtge_Pwr | -0.003 | 0.006 |
|  | (0.609) | (0.399) |
| Polit_pwr | -0.031 | -0.005 |
|  | (0.362) | (0.901) |
| Firm_siz | 0.002 | -0.001 |
|  | (0.609) | (0.869) |
| Levge | -0.142^**^ | -0.022 |
|  | (0.046) | (0.818) |
| RoA | -0.682^*^ | -0.011 |
|  | (0.053) | (0.971) |
| AgE | 0.027^*^ | -0.020 |
|  | (0.094) | (0.574) |
| NegEarn | -0.082^*^ | -0.004 |
|  | (0.087) | (0.919) |
| Big4 | 0.007 | -0.025 |
|  | (0.765) | (0.654) |
| Growth_Re | 0.001 | 0.001 |
|  | (0.697) | (0.833) |
| B_size | 0.001 | 0.000 |
|  | (0.886) | (0.957) |
| B_Ind | -0.002 | 0.034 |
|  | (0.911) | (0.146) |
| Inst_Own | -0.001 | -0.001 |
|  | (0.559) | (0.910) |
| Year & Ind. | Yes | Yes |
| Observations | 520 | 514 |
| A.R. (1) ρ-val. | 0.006 | 0.001 |
| A.R. (2) ρ-val. | 0.117 | 0.547 |
| Hansen J stats p-val. | 0.950 | 0.949 |

*p*-values in parentheses ^*^ *p* < 0.10, ^**^ *p* < 0.05, ^***^ *p* < 0.01

Abernethy, M. A., Kuang, Y. F., & Qin, B. (2015). The influence of CEO power on compensation contract design. *The accounting review, 90*(4), 1265-1306. doi:<https://doi.org/10.2308/accr-50971>

Ahmed, S., & Uddin, S. (2018). Toward a political economy of corporate governance change and stability in family business groups: A morphogenetic approach. *Accounting, Auditing & Accountability Journal*. doi:<https://doi.org/10.1108/AAAJ-01-2017-2833>

Alhadab, M., & Clacher, I. (2018). The impact of audit quality on real and accrual earnings management around IPOs. *The British Accounting Review, 50*(4), 442-461. doi:<https://doi.org/10.1016/j.bar.2017.12.003>

Ali, A., & Zhang, W. (2015). CEO tenure and earnings management. *Journal of Accounting and Economics*

*59*(1), 60-79. doi:<https://doi.org/10.1016/j.jacceco.2014.11.004>

Altarawneh, M., Shafie, R., Ishak, R., & Ghaleb, B. A. A. (2022). Chief executive officer characteristics and discretionary accruals in an emerging economy. *Cogent Business & Management, 9*(1), 2024117. doi:<https://doi.org/10.1080/23311975.2021.2024117>

Altunbaş, Y., Thornton, J., & Uymaz, Y. (2018). CEO tenure and corporate misconduct: Evidence from US banks. *Finance Research Letters, 26*, 1-8. doi:<https://doi.org/10.1016/j.frl.2017.11.003>

Alzoubi, E. S. S. (2018). Audit quality, debt financing, and earnings management: Evidence from Jordan. *Journal of International Accounting, Auditing and Taxation, 30*, 69-84. doi:<https://doi.org/10.1016/j.intaccaudtax.2017.12.001>

Anagnostopoulou, S. C., & Tsekrekos, A. E. (2017). The effect of financial leverage on real and accrual-based earnings management. *Accounting and Business Research, 47*(2), 191-236. doi:<https://doi.org/10.1080/00014788.2016.1204217>

Antonakis, J., Bendahan, S., Jacquart, P., & Lalive, R. (2010). On making causal claims: A review and recommendations. *The leadership quarterly, 21*(6), 1086-1120. doi:<https://doi.org/10.1016/j.leaqua.2010.10.010>

Arellano, M., & Bond, S. (1991). Some tests of specification for panel data: Monte Carlo evidence and an application to employment equations. *The review of economic studies, 58*(2), 277-297. doi:<https://doi.org/10.2307/2297968>

Arellano, M., & Bover, O. (1995). Another look at the instrumental variable estimation of error-components models. *Journal of Econometrics, 68*(1), 29-51. doi:<https://doi.org/10.1016/0304-4076(94)01642-D>

Badolato, P. G., Donelson, D. C., & Ege, M. (2014). Audit committee financial expertise and earnings management: The role of status. *Journal of Accounting and Economics, 58*(2-3), 208-230. doi:<https://doi.org/10.1016/j.jacceco.2014.08.006>

Baker, T. A., Lopez, T. J., Reitenga, A. L., & Ruch, G. W. (2019). The influence of CEO and CFO power on accruals and real earnings management. *Review of Quantitative Finance and Accounting, 52*(1), 1-21. doi:<https://doi.org/10.1007/s11156-018-0711-z>

Berrone, P., Cruz, C., & Gomez-Mejia, L. R. (2012). Socioemotional wealth in family firms: Theoretical dimensions, assessment approaches, and agenda for future research. *Family Business Review, 25*(3), 258-279. doi:<https://doi.org/10.1177/0894486511435355>

Bhandari, A., Mammadov, B., Shelton, A., & Thevenot, M. (2018). It is not only what you know, it is also who you know: CEO Network Connections and Financial Reporting Quality. *Auditing: A Journal of Practice & Theory, 37*(2), 27-50. doi:<https://doi.org/10.2308/ajpt-51821>

Blagoeva, R. R., Mom, T. J., Jansen, J. J., & George, G. (2020). Problem-solving or self-enhancement? A power perspective on how CEOs affect R&D search in the face of inconsistent feedback. *Academy of Management Journal, 63*(2), 332-355. doi:<https://doi.org/10.5465/amj.2017.0999>

Blundell, R., & Bond, S. (1998). Initial conditions and moment restrictions in dynamic panel data models. *Journal of Econometrics, 87*(1), 115-143. doi:<https://doi.org/10.1016/S0304-4076(98)00009-8>

Bouaziz, D., Salhi, B., & Jarboui, A. (2020). CEO characteristics and earnings management: empirical evidence from France. *Journal of Financial Reporting and Accounting*. doi: <https://doi.org/10.1108/JFRA-01-2019-0008>

Bruynseels, L., & Cardinaels, E. (2014). The audit committee: Management watchdog or personal friend of the CEO? *The accounting review, 89*(1), 113-145. doi:<https://doi.org/10.2308/accr-50601>

Chahine, S., Fang, Y., Hasan, I., & Mazboudi, M. (2021). CEO Network Centrality and the Likelihood of Financial Reporting Fraud. *Abacus*. doi:<https://doi.org/10.1111/abac.12219>

Chihua, L., Tseng, Y., & Chen, T.-K. (2016). Top management team expertise and corporate real earnings management activities. *Advances in accounting, 34*, 117-132. doi:<https://doi.org/10.1016/j.adiac.2016.07.007>

Chikh, S., & Filbien, J.-Y. (2011). Acquisitions and CEO power: Evidence from French networks. *Journal of Corporate Finance, 17*(5), 1221-1236. doi:<https://doi.org/10.1016/j.jcorpfin.2011.06.007>

Choi, A., Lee, E. Y., Park, S., & Sohn, B. C. (2021). The differential effect of accrual-based and real earnings management on audit fees: international evidence. *Accounting and Business Research*, 1-37. doi:<https://doi.org/10.1080/00014788.2021.1911779>

Combs, J. G., Ketchen Jr, D. J., Perryman, A. A., & Donahue, M. S. (2007). The moderating effect of CEO power on the board composition–firm performance relationship. *Journal of management studies, 44*(8), 1299-1323. doi: <https://doi.org/10.1111/j.1467-6486.2007.00708.x>

Darouichi, A., Kunisch, S., Menz, M., & Cannella Jr, A. A. (2021). CEO tenure: An integrative review and pathways for future research. *Corporate Governance: An International Review*. doi: <https://doi.org/10.1111/corg.12396>

Dbouk, W., Fang, Y., Liu, L., & Wang, H. (2020). Do social networks encourage risk-taking? Evidence from bank CEOs. *Journal of Financial Stability, 46*, 100708. doi:<https://doi.org/10.1016/j.jfs.2019.100708>

Dechow, P. M., Weili, G., & Catherine, S. (2010). Understanding earnings quality: A review of the proxies, their determinants and their consequences. *Journal of Accounting and Economics, 50*(2-3), 344-401. doi:<https://doi.org/10.1016/j.jacceco.2010.09.001>

Ding, Y., Zhang, H., & Zhang, J. (2007). Private vs state ownership and earnings management: Evidence from Chinese listed companies. *Corporate Governance: An International Review, 15*(2), 223-238. doi:<https://doi.org/10.1111/j.1467-8683.2007.00556.x>

Duru, A., Iyengar, R. J., & Zampelli, E. M. (2016). The dynamic relationship between CEO duality and firm performance: The moderating role of board independence. *Journal of Business Research, 69*(10), 4269-4277. doi:<https://doi.org/10.1016/j.jbusres.2016.04.001>

Fabrizi, M., & Parbonetti, A. (2017). CEO incentives and the trade-off among earnings game strategies. *Academy of Accounting and Financial Studies Journal*

*21*(1), 12-36.

Fama, E. F., & Jensen, M. C. (1983). Separation of Ownership and Control. *The journal of law and Economics, 26*(2), 301-325. doi:<https://doi.org/10.1086/467037>

Fang, M., Francis, B., Hasan, I., & Wu, Q. (2022). External social networks and earnings management. *The British Accounting Review, 54*(2), 101044. doi:<https://doi.org/10.1016/j.bar.2021.101044>

Farag, H., & Mallin, C. (2018). The influence of CEO demographic characteristics on corporate risk-taking: evidence from Chinese IPOs. *The European Journal of Finance, 24*(16), 1528-1551. doi:<https://doi.org/10.1080/1351847X.2016.1151454>

Ferramosca, S., & Allegrini, M. (2018). The complex role of family involvement in earnings management. *Journal of Family Business Strategy, 9*(2), 128-141. doi:<https://doi.org/10.1016/j.jfbs.2018.01.001>

Finkelstein, S. (1992). Power in top management teams: Dimensions, measurement, and validation. *Academy of Management Journal, 35*(3), 505-538. doi:<https://doi.org/10.5465/256485>

Finkelstein, S., & D'aveni, R. A. (1994). CEO Duality as a Double-Edged Sword: How Boards of Directors Balance Entrenchment Avoidance and Unity of Command. *Academy of Management Journal, 37*(5), 1079-1108. doi:<https://doi.org/10.5465/256667>

Florackis, C., & Sainani, S. (2021). Can CFOs resist undue pressure from CEOs to manage earnings? *Journal of Corporate Finance, 67*, 101859. doi:<https://doi.org/10.1016/j.jcorpfin.2020.101859>

Francis, J., Huang, A. H., Rajgopal, S., & Zang, A. Y. (2008). CEO reputation and earnings quality. *Contemporary Accounting Research, 25*(1), 109-147. doi: <https://doi.org/10.1506/car.25.1.4>

Gounopoulos, D., & Pham, H. (2018). Financial expert CEOs and earnings management around initial public offerings. *The International journal of accounting, 53*(2), 102-117. doi:<https://doi.org/10.1016/j.intacc.2018.04.002>

Griffin, P. A., Hong, H. A., Liu, Y., & Ryou, J. W. (2021). The dark side of CEO social capital: Evidence from real earnings management and future operating performance. *Journal of Corporate Finance*, 101920. doi:<https://doi.org/10.1016/j.jcorpfin.2021.101920>

Harris, O., Karl, J. B., & Lawrence, E. (2019). CEO compensation and earnings management: Does gender really matters? *Journal of Business Research, 98*, 1-14. doi:<https://doi.org/10.1016/j.jbusres.2019.01.013>

Healy, P. M., & Wahlen, J. M. (1999). A review of the earnings management literature and its implications for standard setting. *Accounting horizons, 13*(4), 365-383. doi:<https://doi.org/10.2308/acch.1999.13.4.365>

Hill, A. D., Johnson, S. G., Greco, L. M., O’Boyle, E. H., & Walter, S. L. (2021). Endogeneity: A review and agenda for the methodology-practice divide affecting micro and macro research. *Journal of Management, 47*(1), 105-143. doi:<https://doi.org/10.1177/0149206320960533>

Hope, O.-K., Thomas, W. B., & Vyas, D. (2013). Financial reporting quality of US private and public firms. *The accounting review, 88*(5), 1715-1742. doi:<https://doi.org/10.2308/accr-50494>

Hribar, P., & Nichols, C. D. (2007). The use of unsigned earnings quality measures in tests of earnings management. *Journal of Accounting Research, 45*(5), 1017-1053. doi:<https://doi.org/10.1111/j.1475-679X.2007.00259.x>

Hsieh, T.-S., Bedard, J. C., & Johnstone, K. M. (2014). CEO Overconfidence and Earnings Management During Shifting Regulatory Regimes. *Journal of Business Finance & Accounting, 41*(9-10), 1243-1268. doi:10.1111/jbfa.12089

Hsieh, Y.-T., Chen, T.-K., Tseng, Y.-J., & Lin, R.-C. (2018). Top Management Team Characteristics and Accrual-Based Earnings Management. *The International journal of accounting, 53*(4), 314-334. doi:<https://doi.org/10.1016/j.intacc.2018.11.004>

Hu, M., & Gan, S. (2017). Life cycle stage effects, CEO power and internal control quality: evidence from listed family firms in China. *China Journal of Accounting Studies, 5*(2), 211-233. doi:<https://doi.org/10.1080/21697213.2017.1339429>

Jensen, M. C., & Meckling, W. H. (1976). Theory of the firm: Managerial behavior, agency costs and ownership structure. *Journal of Financial Economics, 3*(4), 305-360. doi:<https://doi.org/10.1016/0304-405X(76)90026-X>

Joseph, J., Ocasio, W., & McDonnell, M.-H. (2014). The structural elaboration of board independence: Executive power, institutional logics, and the adoption of CEO-only board structures in US corporate governance. *Academy of Management Journal, 57*(6), 1834-1858. doi:<https://doi.org/10.5465/amj.2012.0253>

Khanna, V., Kim, E. H., & Lu, Y. (2015). CEO connectedness and corporate fraud. *The Journal of Finance, 70*(3), 1203-1252. doi:<https://doi.org/10.1111/jofi.12243>

Koo, K., & Kim, J. (2019). CEO power and firm opacity. *Applied Economics Letter, 26*(10), 791-794. doi:<https://doi.org/10.1080/13504851.2018.1497841>

Kovach, M. (2020). Leader Influence: A Research Review of French & Raven’s (1959) Power Dynamics. *The Journal of Values-Based Leadership, 13*(2), 15. doi:<http://dx.doi.org/10.22543/0733.132.1312>

Krishnan, G. V., Raman, K. K., Yang, K., & Yu, W. (2011). CFO/CEO-board social ties, Sarbanes-Oxley, and earnings management. *Accounting horizons, 25*(3), 537-557. doi:<https://doi.org/10.2308/acch-50028>

Le, H. T. M., Kweh, Q. L., Ting, I. W. K., & Nourani, M. (2020). CEO power and earnings management: Dual roles of foreign shareholders in Vietnamese listed companies. *International Journal of Finance & Economics*. doi: <https://doi.org/10.1002/ijfe.2211>

Leon, L., & ChiiShyan, K. (2017). CEO equity compensation and earnings management: The role of growth opportunities. *Finance Research Letters, 20*, 289-295. doi:<https://doi.org/10.1016/j.frl.2016.10.013>

Lisic, L. L., Neal, T. L., Zhang, I. X., & Zhang, Y. (2016). CEO Power, Internal Control Quality, and Audit Committee Effectiveness in Substance Versus in Form. *Contemporary Accounting Research, 33*(3), 1199-1237. doi: <https://doi.org/10.1111/1911-3846.12177>

Mande, V., & Son, M. (2012). CEO Centrality and Meeting or Beating Analysts’ Earnings Forecasts. *Journal of Business Finance & Accounting, 39*(1‐2), 82-112. doi: <https://doi.org/10.1111/j.1468-5957.2011.02262.x>

Meo, F. D., Lara, J. M. G., & Surroca, J. A. (2017). Managerial entrenchment and earnings management. *Journal of Accounting and Public Policy*

*36*(5), 399-414. doi:<https://doi.org/10.1016/j.jaccpubpol.2017.07.003>

Muttakin, M. B., Khan, A., & Mihret, D. G. (2017). Business group affiliation, earnings management and audit quality: evidence from Bangladesh. *Managerial Auditing Journal*. doi:<https://doi.org/10.1108/MAJ-01-2016-1310>

Muttakin, M. B., Khan, A., & Mihret, D. G. (2018). The effect of board capital and CEO power on corporate social responsibility disclosures. *Journal of Business Ethics, 150*(1), 41-56. doi:<https://doi.org/10.1007/s10551-016-3105-y>

Nickell, S. (1981). Biases in dynamic models with fixed effects. *Econometrica: Journal of the econometric society*, 1417-1426. doi:<https://doi.org/10.2307/1911408>

Nuanpradit, S. (2019). Real earnings management in Thailand: CEO duality and serviced early years. *Asia-Pacific Journal of Business Administration*

*11*(1), 88-108. doi:<https://doi.org/10.1108/APJBA-08-2018-0133>

Oz, I. O., & Yelkenci, T. (2018). Examination of real and accrual earnings management: A cross-country analysis of legal origin under IFRS. *International Review of Financial Analysis, 58*, 24-37. doi:<https://doi.org/10.1016/j.irfa.2018.04.003>

Petrou, A. P., & Procopiou, A. (2016). CEO Shareholdings and Earnings Manipulation: A Behavioral Explanation. *European Management Review, 13*(2), 137-148. doi: <https://doi.org/10.1111/emre.12073>

Priscilla, M., & Siregar, S. V. (2020). The Effect of Top Management Team Expertise on Corporate’s Accrual and Real Earnings Management. *Advanced Issues in the Economics of Emerging Markets*: Emerald Publishing Limited.

Rashid, A. (2016). Managerial ownership and agency cost: Evidence from Bangladesh. *Journal of Business Ethics, 137*(3), 609-621. doi:<https://doi.org/10.1007/s10551-015-2570-z>

Robbins, S. P., & Judge, T. A. (2017). *Organizational behavior, Edition 17, Global edition*. Retrieved from:

Roodman, D. (2009). How to do xtabond2: An introduction to difference and system GMM in Stata. *The stata journal, 9*(1), 86-136. doi:<https://doi.org/10.1177/1536867X0900900106>

Roychowdhury, S. (2006). Earnings management through real activities manipulation. *Journal of Accounting and Economics*

*42*(3), 335-370. doi:<https://doi.org/10.1016/j.jacceco.2006.01.002>

Salehi, M., Bayaz, M. L. D., & Naemi, M. (2018). The effect of CEO tenure and specialization on timely audit reports of Iranian listed companies. *Management Decision*. doi:<https://doi.org/10.1108/MD-10-2017-1018>

Sani, A. u. A., Latif, R. A., & Al-Dhamari, R. A. (2020). CEO discretion, political connection and real earnings management in Nigeria. *Management Research Review, 43*(8), 909-929. doi:<https://doi.org/10.1108/MRR-12-2018-0460>

Sheikh, S. (2019). An examination of the dimensions of CEO power and corporate social responsibility. *Review of Accounting and Finance, 18*(2), 221-244. doi:doi:10.1108/RAF-01-2018-0034

Shiah-Hou, S.-R. (2021). Powerful CEOs and earnings quality. *Managerial Finance*. doi:<https://doi.org/10.1108/MF-07-2020-0363>

Sobhan, A. (2016). Where institutional logics of corporate governance collide: Overstatement of compliance in a developing country, Bangladesh. *Corporate Governance: An International Review, 24*(6), 599-618. doi: <https://doi.org/10.1111/corg.12163>

Strebulaev, I. A., & Yang, B. (2013). The mystery of zero-leverage firms. *Journal of Financial Economics, 109*(1), 1-23. doi:<https://doi.org/10.1016/j.jfineco.2013.02.001>

Tholen, G., Brown, P., Power, S., & Allouch, A. (2013). The role of networks and connections in educational elites’ labour market entrance. *Research in Social Stratification and Mobility, 34*, 142-154. doi:<https://doi.org/10.1016/j.rssm.2013.10.003>

Uddin, S. (2009). Rationalities, domination and accounting control: A case study from a traditional society. *Critical Perspectives on Accounting, 20*(6), 782-794. doi:<https://doi.org/10.1016/j.cpa.2007.11.004>

Ullah, S., Akhtar, P., & Zaefarian, G. (2018). Dealing with endogeneity bias: The generalized method of moments (GMM) for panel data. *Industrial Marketing Management, 71*, 69-78. doi:<https://doi.org/10.1016/j.indmarman.2017.11.010>

Wang, Z., Chen, M. H., Chin, C. L., & , & Zheng, Q. (2017). Managerial ability, political connections, and fraudulent financial reporting in China. *Journal of Accounting and Public Policy*

*36*(2), 141-162. doi:<https://doi.org/10.1016/j.jaccpubpol.2017.02.004>

Wen, Y., Rwegasira, K., & Bilderbeek, J. (2002). Corporate governance and capital structure decisions of the Chinese listed firms. *Corporate Governance: An International Review, 10*(2), 75-83. doi:<https://doi.org/10.1111/1467-8683.00271>

Windmeijer, F. (2005). A finite sample correction for the variance of linear efficient two-step GMM estimators. *Journal of Econometrics, 126*(1), 25-51. doi:<https://doi.org/10.1016/j.jeconom.2004.02.005>

Wintoki, M. B., Linck, J. S., & Netter, J. M. (2012). Endogeneity and the dynamics of internal corporate governance. *Journal of Financial Economics, 105*(3), 581-606. doi:<https://doi.org/10.1016/j.jfineco.2012.03.005>

Yamaguchi, T. (2022). Earnings management to achieve industry-average profitability in Japan. *Asia-Pacific Journal of Accounting & Economics, 29*(2), 402-431. doi:<https://doi.org/10.1080/16081625.2020.1726188>

Zaefarian, G., Kadile, V., Henneberg, S. C., & Leischnig, A. (2017). Endogeneity bias in marketing research: Problem, causes and remedies. *Industrial Marketing Management, 65*, 39-46. doi:<https://doi.org/10.1016/j.indmarman.2017.05.006>
