# Supplementary material for: Do powerful CEOs matter for earnings quality? Evidence from Bangladesh
Source: PLoS One. 2023 Jan 20;18(1):e0276935. doi: 10.1371/journal.pone.0276935 (PMC9858436; doi:10.1371/journal.pone.0276935)
Supplement: Attachment — Submitted filename: Response to Reviewers .docx [file pone.0276935.s003.docx]

**Do powerful CEOs matter for earnings quality? Evidence from Bangladesh**

| **Reviewers’ Comments After First Revision** | **Authors’ Responses** |
| --- | --- |
| **Reviewer #2: PONE-D-22-04720R1** |  |
| **Comments fully addressed, no further comments.** | No further comments by the reviewer. All previous comments are fully addressed. |
| **Reviewer #3:** |  |
| **Authors did not consider any of my comments. They have to acknowledge the reviewer comments to make it publishable.** |  |
| **Original Comments by Reviewer # 3:**  1. First, if authors want to make any additional analysis, then they can make sub-sample of data to better deal with the issue of financing constraints. Gertler and Gilchrest (1993) state that large firms have more external finance premium than smaller firms, which could be due to two reasons: first, large firms have more collateral assets that help them to easily finance their investments and secondly, large firms might be having their business group that helps them to use their own internal capital market. Gertler and Gilchrist (1994) argued that small companies act as a proxy for financially constrained firms because these companies exhibit greater bank dependencies, cannot issue public debt, and face a higher level of idiosyncratic risk. Apart from this, smaller firms are usually younger, with a high level of firm-specific risk and have less collateral, thereby reducing the possibility of attracting external finance. Gupta et el. (2021) also documented that small firms are more financially constrained than large firms. *Here, authors should divide the sample on the basis of firm size (small firms and large firms) to know the how CEO’s Power affect firm earning quality for small firms and large firms as small firms are different than large firms.* | Based on the reviewer's comment, an additional analysis on firm size effect is included in the additional analysis section. The authors divided the sample into two groups (large and small firms), taking the average firm size as the cutoff point for dividing the sample, and then estimated Model 1 to examine whether the effect of powerful CEOs on earnings quality varies between large and small firms. The regression results are shown in Table 7. Table 7 is also attached as an appendix to this file. Please see pages 39–42 of the word-file named “Manuscript” or pages 48–51 of the word-file named “Revised Manuscript with Track Changes”. The write-up is pasted here:  *This study also investigates whether the effect of powerful CEOs on earnings quality varies between large and small firms in Bangladesh. Prior studies suggest that small firms generally face more liquidity constraints than large firms, as these firms have less access to commercial paper and public debt [137-141]. Accordingly, small firms depend on banks and other financial institutions to arrange a significant part of their required funds [138], whereas larger companies are less financially constrained and have fewer issues with money when investing in a project [142]. In addition, small firms face more idiosyncratic risks than large firms [137]. Thus, the behavior of powerful CEOs concerning earnings quality may differ in large and small firms. This study argues that as the scope of fund generation for small firms is relatively narrow, the CEOs of these firms face more pressure to satisfy the debt covenants of banks and financial institutions. These CEOs may be involved in more earnings management practices to reduce the perceived risks of creditors (i.e., the probability of not paying the debt) and to raise additional funds under favorable credit terms [121]. Accordingly, this study predicts that –*  *powerful CEOs in small firms are associated with more earnings management practices than powerful CEOs in big firms.*  *To investigate this issue, this study divides the sample into two groups (large and small firms), taking the average firm size, 21.67, as the cutoff point. Firm-year observations with a mean firm size (log of total assets) of less than 21.67 are classified as small firms, and firm-year observations with a mean firm size equal to or greater than 21.67 are classified as large firms. Then, this study estimates Model 1 for both large and small firms, considering the two earnings quality proxies, DisTAC and DisTA. The estimated results are presented in Table 7.*  *The results in Table 7 show that structural power (Strct_pwr) has negative and statistically significant coefficients at the 5% level for both large and small firms, and across both the earnings quality proxies, DisTAC and DisTA. Expert power (Expt_pwr) has negative coefficients for both large and small firms for the proxy DisTAC. For the proxy DisTA, expert power has a negative coefficient for large firms and a positive coefficient for small firms. The coefficients of expert power are statistically insignificant for large and small firms altogether. Political power has negative coefficients for both large and small firms and across both the earnings quality proxies, DisTAC and DisTA. The coefficients of political power are also statistically insignificant. The rest of the two power dynamics, prestige (Prtge_Pwr) and ownership power (Own_pwr), also suggest an insignificant difference between large and small firms, except for a positive and statistically significant association between ownership power and the earnings quality proxy, DisTAC, for large firms. Thus, overall, these findings imply that, in the context of Bangladesh, the effect of powerful CEOs on earnings quality for large and small firms is identical. One important reason for the insignificant difference between large and small firms to the influence of powerful CEOs is that the capital market of Bangladesh is small and the difference between the listed non-financial firms in terms of firm size or total assets is insignificant. As can be seen from the descriptive statistics, the standard deviation of firm size is very low at 1.52. Also, the mean, median, and third quartile values are 21.67, 21.65, and 22.58, respectively, which are very close to each other.* |
| 1A. Authors should also cite the following studies while discussing about financing constraints in terms of firm size.   a. Gertler, M. and Gilchrist, S. (1993), “The role of credit market imperfections in the monetary transmission mechanism: arguments and evidence”, The Scandinavian Journal of Economics, Vol. 95 No. 1, pp. 43-64. | The referred paper is cited on page 39 under the section ‘Additional analysis’, of the file titled ‘Manuscript’. The citation number for this paper is 138. The sentence that contains the reference is inserted here:  *“This study also investigates whether the effect of powerful CEOs on earnings quality varies between large and small firms in Bangladesh. Prior studies suggest that small firms generally face more liquidity constraints than large firms, as these firms have less access to commercial paper and public debt [137-141].”* |
| b. Gertler, M. and Gilchrist, S. (1994), “Monetary policy, business cycles, and the behavior of small manufacturing firms”, Quarterly Journal of Economics, Vol. 109 No. 2, pp. 309-340. | The referred paper is cited on page 39 under the section ‘additional analysis’, of the file titled ‘Manuscript’. The citation number for this paper is 137. |
| c. Gupta, G., Mahakud, J. and Verma, V. (2021), "CEO's education and investment–cash flow sensitivity: an empirical investigation", International Journal of Managerial Finance, Vol. 17 No. 4, pp. 589-618. | The referred paper is cited on page 39 under the section ‘additional analysis’, of the file titled ‘Manuscript’. The citation number for this paper is 139. The sentence that contains the reference is inserted here:  *“This study also investigates whether the effect of powerful CEOs on earnings quality varies between large and small firms in Bangladesh. Prior studies suggest that small firms generally face more liquidity constraints than large firms, as these firms have less access to commercial paper and public debt [137-141].”* |
| 2. Authors need to write additional hypothesis for small and large size firms analysis. | Formal hypothesis is not explicitly written in this paper, but a prediction is included to test the effect of firm size on CEO power and earnings management practices on page 40 of the file titled “Manuscript”. The prediction is:  *“Powerful CEOs in small firms are associated with more earnings management practices than powerful CEOs in big firms”.* |
| 3. Results of the empirical analysis are clearly presented. But after the implemented changes mentioned for the financial constraints, it should be re-written accordingly. | New empirical results after splitting the sample into large and small firms are presented in Table 7. Table 7 is located on page 41 of the file titled “Manuscript”. Table 7 is also attached as an appendix to this file. |
| 1. Authors should also include the following studies of CEO’s characteristics to strengthen their literature review. 1. Gupta, G., 2022. CEO's age and investment‐cash flow sensitivity. Managerial and Decision Economics. | The referred paper is cited on page 39 under the section ‘additional analysis’, of the file titled “Manuscript”. The citation number for this paper is 141. The sentence that contains the reference is inserted here:  *“This study also investigates whether the effect of powerful CEOs on earnings quality varies between large and small firms in Bangladesh. Prior studies suggest that small firms generally face more liquidity constraints than large firms, as these firms have less access to commercial paper and public debt [137-141].”* |
| 2. Farag, H., & Mallin, C. (2018). The influence of CEO demographic characteristics on corporate risk-taking: Evidence from Chinese IPOs. The European Journal of Finance, 24, 1528–1551 | The referred article is cited on page 15 under the heading “Prestigious CEOs and earnings quality” of the file titled “Manuscript”. The citation number for this article is 90. The sentence that contains the reference is inserted here:  *Further, prestige power induces CEOs to take risky initiatives since their social ties help to get new ideas, protect jobs, and improve the chances of re-employment after the CEO is fired for failed risky strategies [88-90].* |
| 3. Gupta, G. (2021). CEO's educational background, economic policy uncertainty and investment-cash flow sensitivity: Evidence from India. Applied Economics, 1–12. | The referred paper is cited on page 39 under the section ‘additional analysis’, of the file titled “Manuscript”. The citation number for this paper is 140. The sentence that contains the reference is inserted here:  *“This study also investigates whether the effect of powerful CEOs on earnings quality varies between large and small firms in Bangladesh. Prior studies suggest that small firms generally face more liquidity constraints than large firms, as these firms have less access to commercial paper and public debt [137-141]”.* |
| 4. Naheed, R., Jawad, M., Naz, M., Sarwar, B., & Naheed, R. (2021). Managerial ability and investment decisions: Evidence from Chinese market. Managerial and Decision Economics, 42, 985–997. | The referred paper is cited on page 39 under the section ‘additional analysis’, of the file titled “Manuscript”. The citation number for this paper is 142. The sentence that contains the reference is inserted here:  *Accordingly, small firms depend on banks and other financial institutions to arrange a significant part of their required funds [138], whereas larger companies are financially unconstrained and have fewer issues with money when investing in a project [142].* |
| **Reviewer #4:** |  |
| **Thanks for addressing all my comments efficiently.** | No further comments by the reviewer. All previous comments are fully addressed. |

**Appendix**

**Table 7. CEO power dynamics and earnings quality: large versus small firms**

| **Variables** | **Large Firms** | | **Small Firms** | | **Large Firms** | | **Small Firms** | |
| --- | --- | --- | --- | --- | --- | --- | --- | --- |
|  | DisTAC | P-val | DisTAC | P-val. | DisTA | P-val. | DisTA | P-val. |
| L. DisTAC | 0.179^**^ | 0.049 | 0.288^***^ | 0.001 |  |  |  |  |
| L. DisTA |  |  |  |  | 0.281*** | 0.001 | 0.258** | 0.028 |
| Strct_pwr | -0.060^**^ | 0.044 | -0.034^**^ | 0.045 | -0.090** | 0.022 | -0.070** | 0.047 |
| Own_pwr | 0.060^**^ | 0.016 | -0.013 | 0.461 | 0.049 | 0.439 | -0.032 | 0.417 |
| Expt_pwr | -0.036 | 0.155 | -0.024 | 0.248 | -0.032 | 0.338 | 0.013 | 0.730 |
| Prtge_Pwr | -0.001 | 0.862 | 0.006 | 0.417 | 0.015 | 0.234 | -0.005 | 0.639 |
| Polit_pwr | -0.035 | 0.310 | -0.024 | 0.511 | -0.137 | 0.178 | -0.029 | 0.421 |
| Firm_siz | 0.002 | 0.707 | 0.001 | 0.718 | 0.010 | 0.235 | 0.014*** | 0.007 |
| Levge | -0.153^***^ | 0.010 | -0.036 | 0.504 | -0.371* | 0.082 | -0.051 | 0.633 |
| RoA | -0.654^**^ | 0.013 | 0.044 | 0.747 | -0.795 | 0.272 | -0.500 | 0.171 |
| AgE | 0.030^*^ | 0.100 | -0.020 | 0.483 | 0.008 | 0.854 | -0.087* | 0.069 |
| NegEarn | -0.070^*^ | 0.050 | 0.003 | 0.946 | -0.112 | 0.327 | -0.046 | 0.473 |
| Big4 | 0.014 | 0.523 | -0.007 | 0.790 | -0.006 | 0.900 | 0.092 | 0.161 |
| Growth_Re | 0.001 | 0.497 | 0.001 | 0.432 | -0.001 | 0.540 | 0.001 | 0.173 |
| B_size | 0.001 | 0.984 | 0.002 | 0.769 | 0.005 | 0.738 | -0.004 | 0.639 |
| B_Ind | -0.001 | 0.970 | 0.026 | 0.107 | 0.032 | 0.240 | 0.056* | 0.096 |
| Inst_Own | -0.001 | 0.657 | 0.001 | 0.626 | -0.002* | 0.096 | 0.001 | 0.386 |
| F-stat | 17.94*** | 0.001 | 33.31*** | 0.001 | 15.35*** | 0.001 | 17.14*** | 0.001 |
| Year & Ind. Dum. | Yes |  | Yes |  | Yes |  | Yes |  |
| Observations | 520 |  | 514 |  | 520 |  | 514 |  |
| A.R. (1) ρ-val. | 0.006 |  | 0.001 |  | 0.048 |  | 0.002 |  |
| A.R. (2) ρ-val. | 0.125 |  | 0.989 |  | 0.468 |  | 0.508 |  |
| Hansen J stat p-val. | 0.891 |  | 0.960 |  | 0.852 |  | 0.980 |  |

GMM estimation with Windmeijer [31] corrected standard errors. Significant values of 1%, 5%, and 10% are indicated by the symbols ***, **, and *, respectively.
